# Estimating the Population Size of Female Sex Workers in Zimbabwe: Comparison of Estimates Obtained Using Different Methods in Twenty Sites and Development of a National-Level Estimate

**DOI:** 10.1097/QAI.0000000000002393

**Published:** 2020-05-05

**Authors:** Elizabeth Fearon, Sungai T. Chabata, Sitholubuhle Magutshwa, Tendayi Ndori-Mharadze, Sithembile Musemburi, Henry Chidawanyika, Absolom Masendeke, Sue Napierala, Elizabeth Gonese, Amy Herman Roloff, Beth A. Tippett Barr, Peter H. Kilmarx, Ramona Wong-Gruenwald, Samson Chidiya, Mutsa Mhangara, Dagmar Hanisch, Jessie K. Edwards, Brian Rice, Isaac Taramusi, Tendai Mbengeranwa, Portia Manangazira, Owen Mugurungi, James R. Hargreaves, Frances M. Cowan

**Affiliations:** a Department of Global Health and Development, London School of Hygiene and Tropical Medicine, London, United Kingdom;; b Centre for Sexual Health and HIV/AIDS Research Zimbabwe, Harare, Zimbabwe;; c RTI International, Harare, Zimbabwe;; d Women's Global Health Imperative, RTI International, San Francisco, CA;; e Division of Global HIV and Tuberculosis, U.S. Centers for Disease Control and Prevention, Harare, Zimbabwe;; f Gesellschaft für Internationale Zusammenarbeit, Eschborn, Germany;; g United States Agency for International Development, Harare, Zimbabwe;; h United Nations Population Fund, Harare, Zimbabwe;; i Department of Epidemiology, University of North Carolina at Chapel Hill, Chapel Hill, NC;; j Department of Public Health, Environments and Society, London School of Hygiene and Tropical Medicine, London, United Kingdom;; k National AIDS Council, Harare, Zimbabwe;; l AIDS and TB Programme, Ministry of Health and Child Care, Harare, Zimbabwe; and; m Department of International Public Health, Liverpool School of Tropical Medicine, Liverpool, United Kingdom.

**Keywords:** HIV, sex workers, women, surveillance, epidemiology, Zimbabwe

## Abstract

National-level population size estimates (PSEs) for hidden populations are required for HIV programming and modelling. Various estimation methods are available at the site-level, but it remains unclear which are optimal and how best to obtain national-level estimates.

**Setting:**

Zimbabwe.

**Methods:**

Using 2015–2017 data from respondent-driven sampling (RDS) surveys among female sex workers (FSW) aged 18+ years, mappings, and program records, we calculated PSEs for each of the 20 sites across Zimbabwe, using up to 3 methods per site (service and unique object multipliers, census, and capture-recapture). We compared estimates from different methods, and calculated site medians. We estimated prevalence of sex work at each site using census data available on the number of 15–49-year-old women, generated a list of all “hotspot” sites for sex work nationally, and matched sites into strata in which the prevalence of sex work from sites with PSEs was applied to those without. Directly and indirectly estimated PSEs for all hotspot sites were summed to provide a national-level PSE, incorporating an adjustment accounting for sex work outside hotspots.

**Results:**

Median site PSEs ranged from 12,863 in Harare to 247 in a rural growth-point. Multiplier methods produced the highest PSEs. We identified 55 hotspots estimated to include 95% of all FSW. FSW nationally were estimated to number 40,491, 1.23% of women aged 15–49 years, (plausibility bounds 28,177–58,797, 0.86–1.79%, those under 18 considered sexually exploited minors).

**Conclusion:**

There are large numbers of FSW estimated in Zimbabwe. Uncertainty in population size estimation should be reflected in policy-making.

## INTRODUCTION

Female sex workers (FSW) in sub-Saharan Africa carry a heavy burden of HIV infection.^1^ Commercial sex plays an important role in driving and sustaining the epidemic, even in generalized epidemics.^2,3^ To support HIV prevention and care programs, resource allocation, and policy-making, estimates of the number of FSW and the locations where they work and access services are required.

A variety of methods are available to estimate the size of stigmatized and/or criminalized populations,^4^ but there is no gold standard. Uncertainty is high, and there is limited evidence as to systematic biases by method.^5^ Further challenges include heterogeneity in types and volume of sex work that women selling sex are involved in.

Although methods for population size estimation have primarily been developed for the level of an individual city, town, or other hotspot (a site where sex work is concentrated), national-level estimates are required for planning.^6^ Mathematical models of HIV transmission are often fitted to country-level data and play a central role in prioritization and funding decisions. Despite this, the methods by which national estimates are derived from sites are often not systematically collected or well-documented.^7,8^

Across sub-Saharan Africa, the combined percentage of the female population engaged in sex work (ages 18–49) or subjected to commercial sexual exploitation (ages 15–17) has been estimated to range from 0.76 to 1.0% in South Africa,^9^ to 2% in Cameroon^10^ and 5% among the urban female Kenyan population.^11^ A review of studies from 1995 to 2005 found that between 0.4 and 4.3% of the 15–49-year-old female population across urban areas of sub-Saharan Africa were estimated to be engaged in sex work.^12^

Zimbabwe has until now lacked a documented national-level estimate of the number of FSW. From 2015 to 2017, we obtained population size estimates (PSEs) of FSW at 20 sites around the country using, in most cases, multiple methods per site. Here, we compare PSEs obtained using different methods within the same site to assess the potential for systematic bias by method. We then describe the systematic matching and stratification approach taken in using these 20 site estimates to develop a national-level estimate.

## METHODS

We used 4 types of data collected from 20 sites across Zimbabwe to obtain site PSEs. We describe first the data; then the 4 methods we used to obtain site PSEs; then our approach to comparing estimates obtained from different PSE methods within each site; and finally, our approach to using the site PSEs to obtain a national-level estimate of the number of FSW.

### Data

We used of 4 types of data in estimating FSW population sizes: (1) respondent-driven sampling (RDS) survey data; (2) social and geographic mapping data; (3) sex worker program data; and (4) population census data.

#### Respondent-Driven Sampling Surveys

We conducted 20 RDS surveys^13–15^ at 20 sites across the country between September 2015 and May 2017, using near-identical protocols. For the 2 largest sites and one small one, FSW population size estimation was a primary aim of the study for which the data were collected. The other sites were selected according to other study aims, including inclusion in a cluster randomized trial of an enhanced FSW intervention,^16^ and as assessments of an FSW program.^17^ For each site RDS survey, 4 to 20 initial “seed” participants were recruited and selected to reflect different ages, sex work types, sub-communities and neighborhoods, and not specifically drawn from FSW program attendees, (program described further below). Each woman was given 2 coupons to refer 2 peers who met the survey eligibility criteria and whom she knew (defined as knowing each other's names), who upon recruitment, consented to completion of a questionnaire and blood samples for HIV testing, until the sample size was reached (5–7 recruitment waves per site, discounting seeds). Eligibility criteria were aged at least 18 years, resident or working in the site for at least 6 months (1 month for 4 of the surveys) and having exchanged sex for money or gifts in the previous 30 days. Women received US$5 remuneration for participation and US$2 for each peer recruited.

The sample sizes for the RDS surveys were based on different considerations reflecting the primary study aims for which they were conducted, including the precision of HIV prevalence estimates, power for a cluster-randomized trial that included 14 of the sites,^18^ and in the case of the 2 largest cities of Harare and Bulawayo, specifically on returning reasonable confidence intervals around the PSEs obtained.^19^ Final sample sizes ranged from 200 FSW in a small site to 808 in Bulawayo and 1497 in Harare, (total n = 6248 across 20 sites, with further detail about 16 of these site surveys available elsewhere^17,20^).

We used RDS-II weighting^14^ with network size determined by asking each woman how many other FSW she knew at the site (ie, she knew their name and they knew her) who met the eligibility criteria, who she had seen in the previous month and who she would consider recruiting to the study. For each site, we investigated whether key RDS assumptions seemed to have been met,^21^ reported elsewhere for some sites,^16,17^ and specifically as pertaining to potential biases in the PSEs for all sites in Supplemental Digital Content (see Appendix 1, http://links.lww.com/QAI/B476). We used the RDS: Respondent-Driven Sampling package^22^ for R version 3.3.2.^23^

#### Mapping Data

At each site directly before each survey, we first conducted social and geographical mapping,^24^ identifying sex workers via key informants (eg, health staff, bartenders, other FSW). We asked what they knew about sites where FSW congregated or found clients in the locality, using this information to make a list of all sex work venues at the site. This information informed seed participant selection for the RDS surveys.

#### Sisters with a Voice Program Visit Data

Eighteen of the 20 sites with RDS surveys were also served by the “Sisters with a Voice” program (“Sisters”), which provides sexual and reproductive health services for FSW at 36 sites across all provinces of Zimbabwe. These sites were added by the program over time from 2009 onward, because they were assumed to have large numbers of FSW requiring services. Each woman attending Sisters is given a unique identification number recorded at each visit. Sisters is specifically aimed at sex workers and it is unlikely that women attending are not engaged in selling sex, given the stigma this entails.^25^

#### Zimbabwe 2012 National Census

We used population denominators of the female population, available as those aged 15–49 years, from the latest (2012) Zimbabwe national census^26^ in calculating the percentage of women engaged in sex work at each site.

### Individual Site PSE Methods

We used 4 PSE methods, described below, across the 20 sites. Four sites used only one method, 2 sites used 2 methods, and 14 sites used 3 methods.

#### Service Multiplier Method

We counted the number of women uniquely identified in Sisters clinic visit records for each site in a reference period of 6 months before the survey (4 sites) or 12 months prior (14 sites). The estimated population size was this count M divided by the RDS-II weighted proportion of women in the site survey [survey and clinic catchment areas correspond, question wording Supplemental Digital Content (see Appendix 2, http://links.lww.com/QAI/B476)], who reported attending the Sisters clinic during the reference period P, meaning that PSE = M/P. We used the Delta method to obtain 95% confidence intervals as recommended, reflecting both variance from the RDS-estimated proportion and in the count of program attenders.^19,27^

#### Unique Object Multiplier Method (UOMM)

The unique object multiplier method (UOMM) works along the same principles as the service multiplier method (SMM)^4^ and has been used to estimate the number of FSW in other populations.^28,29^ A known number of recallable objects are distributed in the target population shortly before a representative survey. These objects are equivalent to the known number of individuals visiting a service within a given reference period in the SMM, M, described above, but the method can be used in areas lacking a FSW service. At the survey, participants are asked whether they received one of the objects, and the number of objects are then divided by the proportion of the target population reporting that they have received one, P. In our study, a known number of wristbands were distributed ahead of the RDS survey to eligible FSW identified at areas/venues identified during the mapping phase. RDS seeds were not given wristbands to distribute to keep this process independent from survey recruitment. We divided the number of wristbands distributed at each site M by the RDS-II weighted proportion of women who reported receiving one in the survey P, to obtain PSE = M/P. The 95% confidence intervals were calculated as above for the SMM.

#### Capture-Recapture

Our capture-recapture (CC) method used one capture and 2 recaptures, as in other FSW studies.^4,30^ The capture was done on Friday night, when teams of 2 survey assistants and a peer educator visited areas/venues identified through mapping to distribute enumeration cards to women meeting the eligibility criteria. Enumeration cards were printed in duplicate and color-coded for 3 different enumeration days, with a serial number such that one copy was retained by the survey team and the other given to the participant. This exercise was repeated on another 2 consecutive Friday nights with women who reported to have been enumerated during the previous exercises identified as recaptures. We used the Schnabel index such that 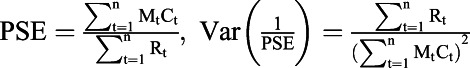 and 95% CI of 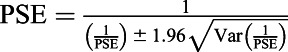
where: 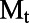 = the total number of FSW who previously received a unique object at time t, 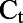 = the number of FSW found at time t, R = the number of recaptures, and t = an individual sample period.

#### Census

In 2 small sites, teams of 6 survey assistants counted FSW on one high-activity night at each of the sex work areas/venues identified during mapping, and the sum in the site was used as the PSE. The census PSEs lacked variance estimates.

### Methods Assessment and Comparison

The SMM was used in 18 sites, the UOMM in 16 sites, CC in 14 sites, and census in 2 sites. We compared PSEs and the extent to which the 95% confidence intervals for the PSE overlapped for each method within 16 sites. To assess the extent to which PSEs differed by method within sites, we calculated the mean and median absolute and relative differences for each within-site pair of methods.

### Methods for Obtaining a National-Level PSE

To review PSE methods, individual site estimates, and agree and inform an approach to formulating a national size estimate, a 2-day workshop was convened [further detail Supplemental Digital Content (see Appendices 3 and 4, http://links.lww.com/QAI/B476)]. Participants included Ministry of Health and Child Care, National AIDS Council, funder, researcher, and FSW program staff and stakeholders. The 20 individual site PSEs described above, 18 of which were from sites with the Sisters program, were made available to the workshop.

#### Sampling Frame

Sisters sites were added by the program on the basis of perceived need, (ie, where it was believed by national and provincial stakeholders and program staff that there were large numbers of FSW present) rather than by random selection, making it implausible to apply prevalence of sex work found in these sites to the country as a whole. We therefore generated a list of all sites with likely concentrations of sex work among women in Zimbabwe, defined as “hotspots,” as our sampling frame. This list included all 36 Sisters sites, as well as other additional sites identified at the workshop. We identified these additional likely sex work hotspots through a structured session with workshop participants, using a map and considering each province in turn, and reaching consensus on additional suggested sites.

#### Hotspot Strata

At the workshop, hotspots that were similar to each other in the proportion of adult women engaged in sex work were matched into strata, based on expert opinion and considering: the number of Sisters program attendees from January–March 2017 where applicable; population size; province; whether urban or rural; and type of site. The latter equated to the dominant economic activity, including mining, transport hub, army base, tourism, provincial capital, and “growth-points.” Zimbabwean growth-points are small rural towns targeted for economic development and service delivery, with the aim of decentralizing the economy away from urban centres.^31^ Workshop participants brought their expertise in HIV epidemiology and experience delivering FSW programs to bear. Each stratum contained at least one of the 20 sites where we had estimated population size directly.

#### Adjustment for FSW Working Outside of Hotspots

Participants were then guided in a discussion to reach consensus on what proportion of all FSW in Zimbabwe would be present in these hotspots (Sisters sites plus additional suggestions), and what proportion may be working outside them.

#### Extrapolation

Using denominators from the Zimbabwe 2012 census, we converted PSEs to prevalences of sex work, (commercial sexual exploitation for those under 18 years), among female 15–49-year-olds in each site. Using maps of census wards, Sisters program staff defined catchment areas for each site. We took the median PSE in sites where we had used more than one method because we lacked evidence to weight one method over another and this approach has been taken in other PSE studies.^32^ Within strata, we took the median prevalence across sites with direct estimates, and applied this to all sites in the stratum, using the census population figures to convert this back to a number of FSW per site. Where sites had a direct PSE, we used the median direct estimate rather than applying the stratum prevalence-based estimate. We then summed the directly estimated and extrapolated PSEs across all hotspots, and applied a final correction reflecting the additional proportion of FSW that had been estimated by the workshop participants to be working outside hotspots.

For plausibility bounds, a term used to describe high and low estimates that are partly, but not entirely statistically based and used in other PSE studies,^27,32,33^ we repeated the process used to reach the point estimate, but took the lowest and highest 95% CI bounds from PSEs obtained for each site instead of the median PSE to include all uncertainty, other than biases that cannot be easily quantified.

### Ethics

We conducted the studies from which data were drawn with ethical approval from Medical Research Council of Zimbabwe and the Research Council of Zimbabwe, University College London, and the London School of Hygiene and Tropical Medicine. One study including 4 sites was reviewed according to the U.S. Centers for Disease Control and Prevention (CDC) human research protection procedures and was determined to be research, but CDC was not engaged. National extrapolation was approved by London School of Hygiene and Tropical Medicine. We do not report individual site names except for the largest cities because the stakeholders' workshop considered this information sensitive and potentially harmful to local FSW.

## RESULTS

### Individual Site PSEs

Site PSEs ranged from 132 (census method) to 12,863 (95% CI: 10,657 to 15,068), Site 1, Harare (SMM), Table 1. Only 3 sites were estimated to include more than 1000 FSW. We report on possible biases in site PSEs in Supplemental Digital Content (see Appendix 1, http://links.lww.com/QAI/B476).

**TABLE 1.**
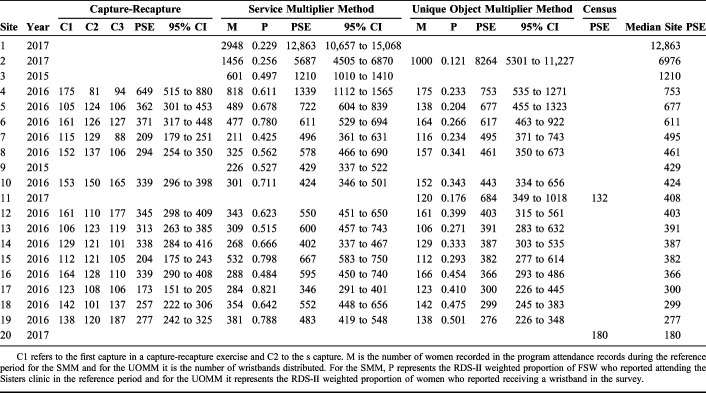
Population Size Estimates (PSEs) Using Four Methods Across 20 Sites With Direct Estimates

### Comparison Across Methods

The SMM produced the highest estimate in 12/16 sites. The UOMM gave the highest estimate in 4 sites. Overall, the SMM resulted in the highest PSEs, a mean of 32% higher than UOMM (absolute number 316 FSW different) and mean of 63% higher than CC (Table 2). On average (mean), the UOMM was 27% higher than the CC estimates, and 135% higher than the census estimate, although the latter comparison existed for only one site.

**TABLE 2.**
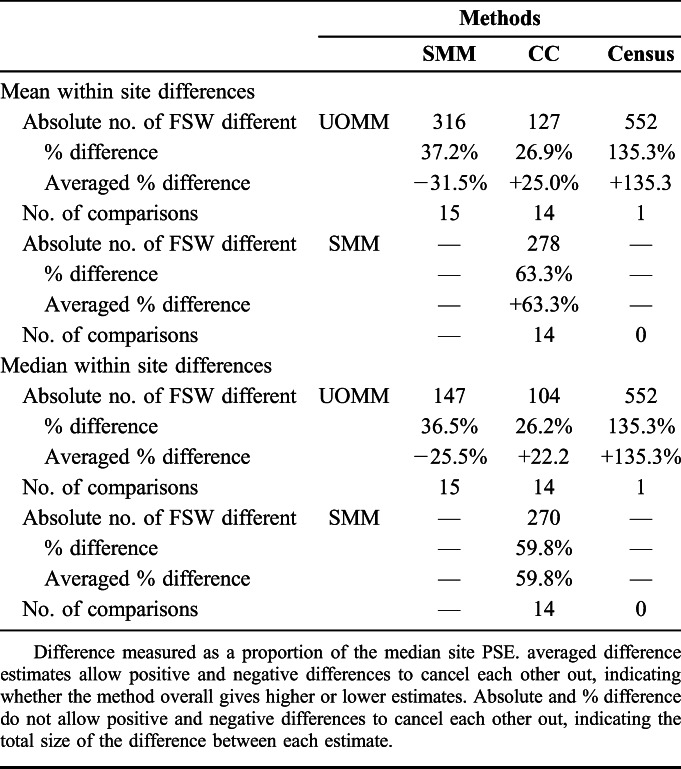
Mean and Median Within-Site Methods Differences Between Estimates Derived From Each PSE Method (Absolute and Relative to Median Site Estimate)

**FIGURE 1.**
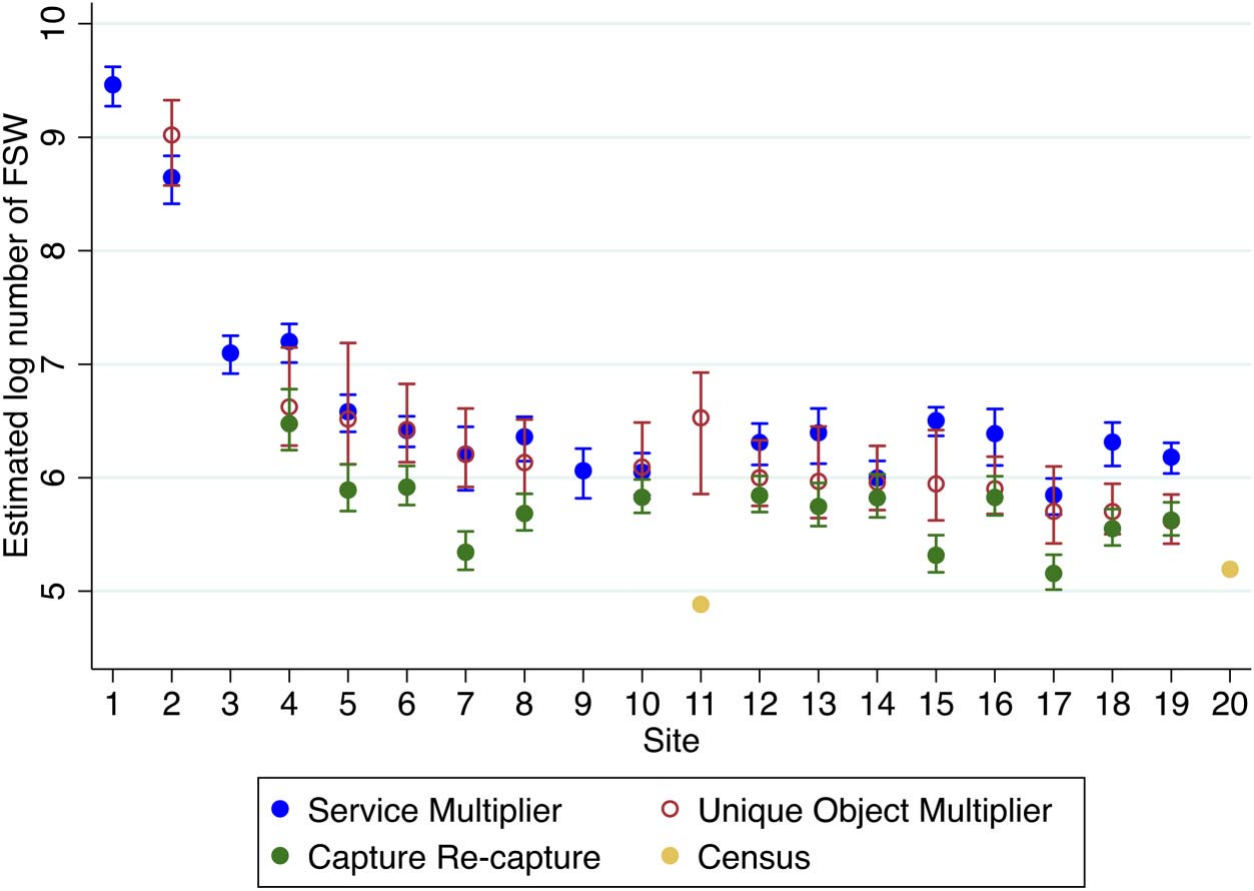
Log population size estimates and 95% confidence intervals for each of the 20 sites by method.

In all 14 sites using 3 PSE methods, the 95% confidence intervals of at least 2 methods overlapped (Fig. 1). In 3 sites, the intervals for each PSE method overlapped, with a further 5 being within the same range. In 4 sites, the SMM and UOMM estimates overlapped, whereas the CC was lower, and in 2 sites the UOMM and CC estimates overlapped, whereas the SMM was higher.

### Hotspot Strata

In addition to the 36 Sisters program sites, the stakeholder consultation suggested a further 19 possible hotspot sites, totaling 55 nationally. Stakeholders estimated that 95% of all FSW in Zimbabwe would be included in these 55 sites.

We identified 10 hotspot strata determined by stakeholders to be similar in their prevalence of sex work. These ranged in size from 12 sites (Strata 4), to 3 strata of only one site each, which were perceived to be singular in their characteristics (all cities), and including Bulawayo and Harare (Table 3).

**TABLE 3.**
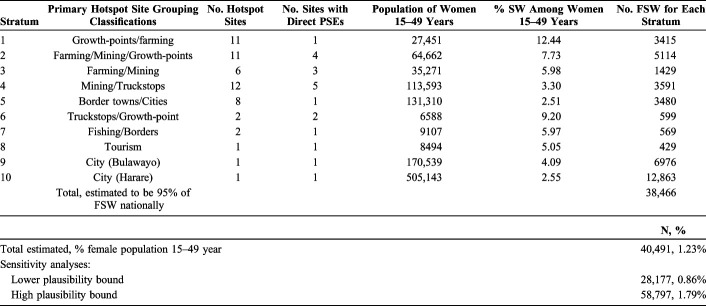
Percentage of Female Sex Workers Among Women Aged 15–49 years Resident in Sites Within the Following Strata

The sites with the highest estimated prevalence of sex work, 12.4%, were those in Stratum 1, which included 11 hotspots whose denominator population of females aged 15–49 years totaled 27,451 (median 2102 per site). These small sites were primarily rural growth-points and farming economies, only one of which had a direct PSE. The prevalence of sex work in other hotspot site strata ranged from 2.51% in border towns/cities to 9.20% in 2 truckstop/growth-point sites.

**FIGURE 2.**
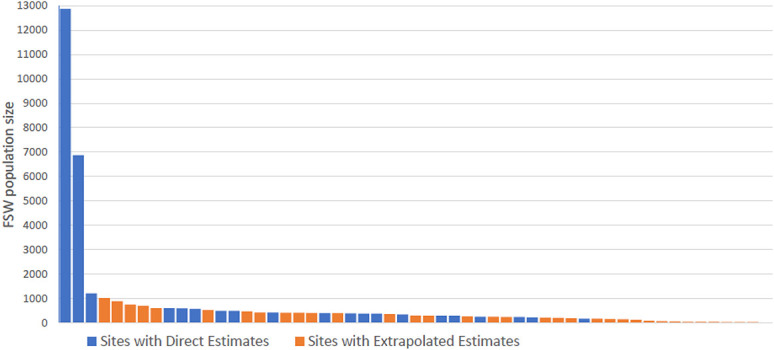
Distribution of the estimated (directly estimated and extrapolated) size of the female sex worker population at 55 hotspot sites.

The distribution of FSW PSEs among hotspots was highly skewed. Most FSW were estimated to be found in a small number of larger sites, the top 3 of which were sites with directly estimated population sizes (Fig. 2), accounting for an estimated 21,049 FSW.

### National PSE

The sum of all PSEs across each of the 55 sites came to 38,466. Estimated to represent 95% of all FSW, this made the national-level estimate 40,491 or 1.23% of the female population aged 15–49 years. Lower and higher plausibility bounds were estimated as 28,177 (0.86%) to 58,797 (1.79%).

## DISCUSSION

We present the first national PSE of FSW in Zimbabwe, now adopted into national policy-making, of 40,491 FSW (plausibility bounds 28,177–59,797), with site level PSEs ranging from 180 (census method) in a rural farming area to 12,863 (median across methods) in Harare. We found individual site PSEs to vary considerably within sites and across methods, with census and CC methods tending to be lower than those obtained via multiplier methods. Our estimates inform HIV modelling, and the national and individual site PSEs provide denominators for monitoring and evaluation using program records, (increasingly requested by funders^34^), and provide a basis for geographically targeting FSW services, appropriately considering uncertainty in estimation, given the considerable variance across methods that we and others have found.^34^

A national prevalence of 1.23% of current sex work among the female population aged 15–49 years and urban prevalences from Harare (2.55%) and Bulawayo (4.09%) are comparable to other regional findings.^10–12^ Most FSW were estimated to be in a few large sites, although the presence of FSW at sites across the country, alongside high incidence and prevalence of HIV in this population,^20,35–37^ indicates the need for a national program and service provision in Harare, Bulawayo, and other urban areas.

Defining the FSW population is challenging: different studies and models define FSW differently, explicitly, or implicitly. Transmission modelling focuses on rates of partner change because this is what drives HIV incidence; programs may be most interested in the number of women who are likely to be served by a sex worker-friendly clinic in a given area. Women recruited into RDS surveys and those attending Sisters are likely to be those who self-define and are defined by their peers as being sex workers. There are likely women who have high rates of partner change and sexual exchange, but who would not regard themselves or be regarded by peers as FSW and would be less likely to be included in our estimates. The evaluation of Zimbabwe's National Behavior Change Program survey in 2011/2012 found that 1.3% of women reported at least 2 transactional sex partners in the past 6 months.^38^ A mathematical model of HIV in Zimbabwe has estimated that there were 127,385 (lower and upper bounds 60,225–200,020) adult women in Zimbabwe who had had at least 3 condomless sex partners in any given 3-month period in the previous year.^39^ It is plausible that the women who were counted as FSW in our study are a subset of the group estimated in the model. Exactly how these different populations relate to and overlap with each other, what their varying service needs are, and how services should be provided, requires further understanding.

Our study contributes to evidence about biases of different size estimation methods.^5^ We found that CC and census methods gave lower PSEs than the multiplier methods, whereas a review of previous studies did not find multiplier estimates to be consistently high or low.^5^ Both CC and census could miss women not present at the area/venue at the time of counting. The SMM assumes that the list of program attendees is independent from the estimation of the proportion of women attending the program. We did make efforts to ensure that this was the case, but found evidence for nonconvergence and possible seed dependence of program attendance and wristband receipt in some sites (see Appendix 1, Supplemental Digital Content, http://links.lww.com/QAI/B476), in all but one case likely over-estimating P, although this would have under-estimated population size, whereas we found the SMM and UOMM estimates to be highest.

### Strengths and Limitations

We used many site PSEs obtained using different methods to develop our national-level estimate, using a systematic approach in collaboration with programmers and policy-makers. We used high-quality survey and program data sources, the latter of which underwent a de-duplication exercise in 2017. We investigated potential biases due to unmet RDS assumptions. For Harare and Bulawayo, we sought to limit random error by basing our RDS survey sample size calculations on size estimation requirements.^19^

Multiple methods are recommended to mitigate biases across methods, but only one method was possible to implement in Harare, the largest site. The age range did not match up exactly between our RDS surveys (18 years and older), the Sisters services data (no age criteria), and the census population denominator of 15–49 years. The census was conducted in 2012 while site estimates date from 2015 to 2017 and projections suggest that the 15–49 population has grown by 3.27% annually (not gender-disaggregated or available by ward).^40^ It is also possible that some women could have migrated between sites and this study does not account for movement in and out of sex work.^41^ Although it is possible that we missed some hotspots from our list, given the distribution of site PSEs, it is unlikely that they would have a large impact on the national estimate. We also caution that the prevalence of sex work estimates for each hotspot strata should not be interpreted to apply to all rural growth-points, or all farming communities, for example.

Our method of reaching a national-level estimate relied on expert opinion for estimating total coverage of the FSW in hotpots and in matching sites into strata, rather than other Bayesian,^42,43^ or missing data-based approaches.^44^ Our approach was chosen because of the large number of site PSEs, nonrandomly selected and which did not map to administrative units with available contextual variables. It utilizes expert experience, but is consequently not completely transparent and means that the statistical properties of the estimate are unknown.

### Conclusions and Recommendations

We provide further evidence to support the recommendation that multiple PSE methods are used to obtain site-level PSEs, preferably those using different data sources and making different assumptions. We recommend that a transparent and documented approach be used whenever site-level PSEs are used to inform national-level estimations, and we recommend our method for obtaining a national level PSE when there are: a large number of high-quality PSEs across diverse sites; if these sites are “hotspots” rather than randomly selected areas, as estimates utilizing programmatic often are; and where the hotspot borders do not map well to administrative units with contextual information that could be utilized in a fully statistical approach. It is essential that uncertainty of PSEs, particularly when extrapolated to larger or smaller areas than those in which estimation was originally conducted, is considered when policy, programming, and resource allocation decisions are based on these estimates. If program targets and funding are tied to PSEs and those PSEs are in fact under-estimated, resources allocated to HIV prevention and treatment will be inadequate. Conversely, if the PSE has been over-estimated, HIV and sexual health programs may be tasked with engaging FSW who do not exist.

Our findings indicate that FSW remain a high-priority population for HIV programming efforts in Zimbabwe, given the large size of this population, their high HIV incidence and prevalence estimates,^16,35,37^ and involvement in HIV transmission.

## Supplementary Material

SUPPLEMENTARY MATERIAL
