# Supplementary material for: Estimating the Population Size of Female Sex Workers in Zimbabwe: Comparison of Estimates Obtained Using Different Methods in Twenty Sites and Development of a National-Level Estimate
Source: J Acquir Immune Defic Syndr. 2020 May 5;85(1):30–8. doi: 10.1097/QAI.0000000000002393 (PMC7417013; doi:10.1097/QAI.0000000000002393)
Supplement: SUPPLEMENTARY MATERIAL [file qai-85-30-s001.pdf]

Appendix 1: Assessments of site-based PSE's

Rationale and Methods

Each population size estimation method makes a number of assumptions, as does respondent driven sampling(1, 2). Some of these assumptions can be investigated for possible biases in the estimate of P (proportion attending clinic or receiving a wristband in the RDS surveys) and implications for the PSE qualitatively assessed. For multiplier methods, the two data sources are assumed to be independent. In the case of the service multiplier method (SMM), this means that women attending the programme should not disproportionately have been more likely to be recruited into the survey, and for the unique object multiplier method (UOMM), that the process of distributing wristbands was independent of survey recruitment. While we made efforts to keep these processes separate (e.g. not recruiting seeds based on programme attendance or distributing wristbands directly to RDS seeds) we graphically examined the convergence of the estimate that measured programme attendance or wristband receipt in the RDS survey over sample accumulation to judge whether there was evidence that the final estimate was likely still dependent on seed characteristics or whether the estimate appeared to have stabilized prior to final sample size ('reasonable convergence'). If the estimate had not converged, this could also have indicated that it was too low or high(3) and therefore that our resulting PSE was too high or too low. We also examined recruitment homophily by programme attendance and wristband receipt (the tendency for women to recruit others like themselves on the basis of a given characteristic) and the ratio of the mean network size of those who did attend or receive a wristband to those who did not, a difference that is accounted for in the weighting but which explains a discrepancy between the unweighted and weighted findings.

Capture-recapture methods assume the 'captures' are independent from each other which is difficult to ensure or assess in practice, and assumes limited mobility of FSW to and from the site between captures. The census methods assume that women counted at sites are indeed sex workers.

We used the RDS package(4, 5) for R statistical software version 3.3.2.(5)

Findings

Convergence plots

Site 1

SMM (Attended Sisters)

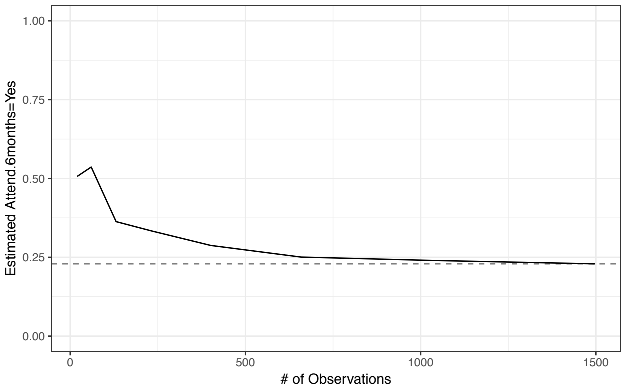

Site 2

SMM (Attended Sisters)

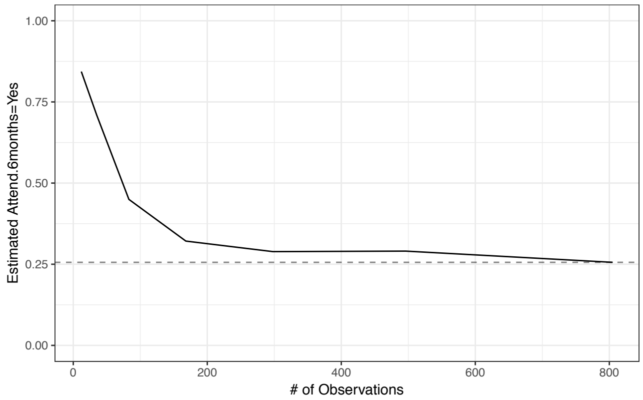

UOMM (Received Wristband)

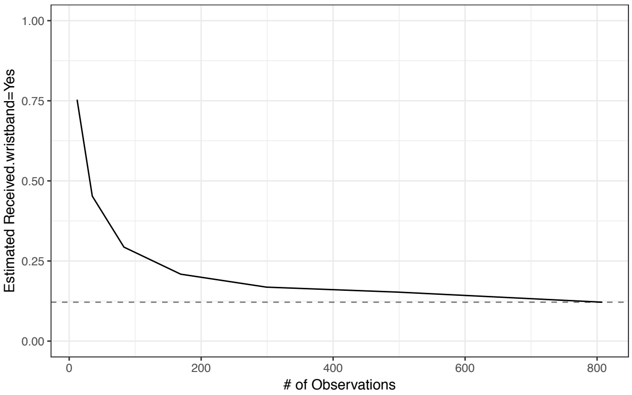

Site 3

SMM (Attended Sisters)

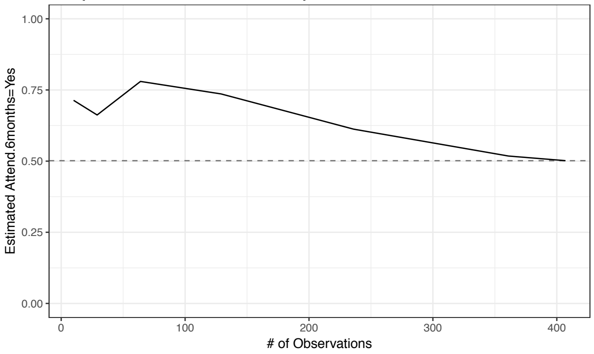

Site 4

SMM (Attended Sisters)

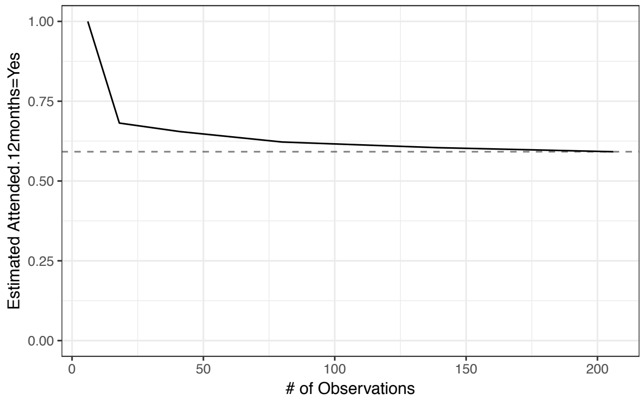

UOMM (Received Wristband)

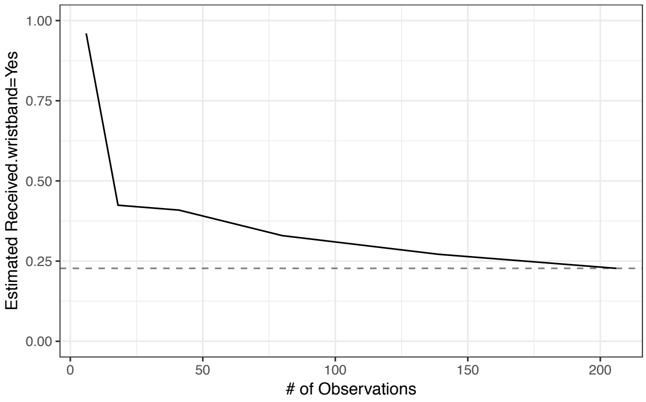

Site 5

SMM (Attended Sisters)

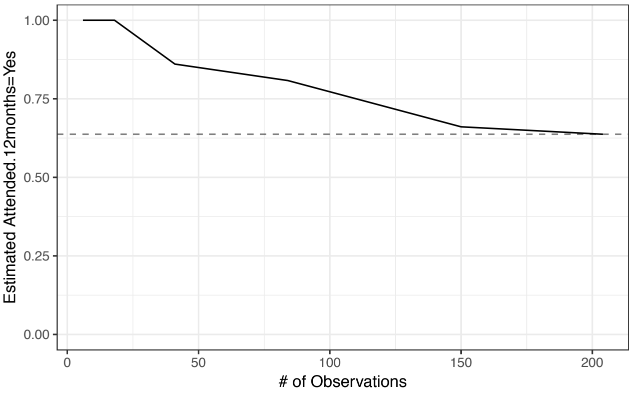

UOMM (Received Wristband)

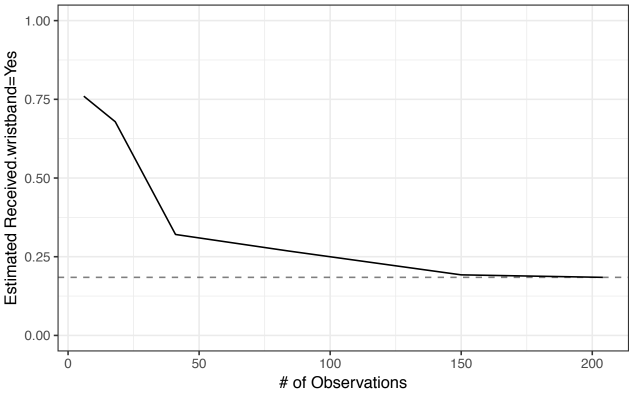

Site 6

SMM (Attended Sisters)

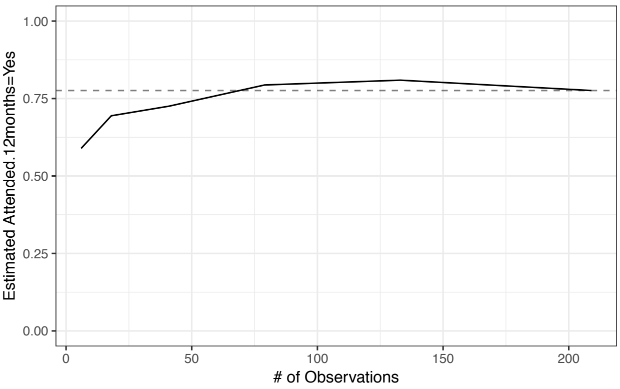

UOMM (Received Wristband)

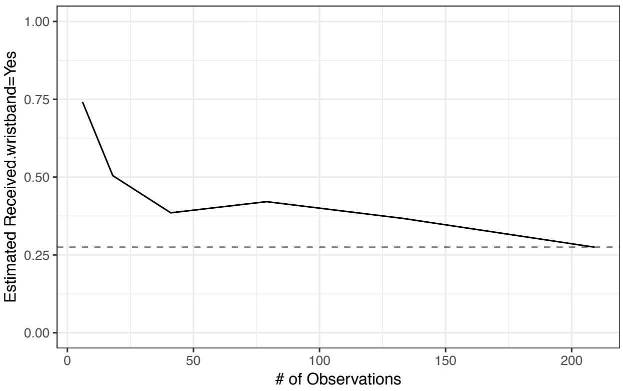

Site 7

SMM (Attended Sisters)

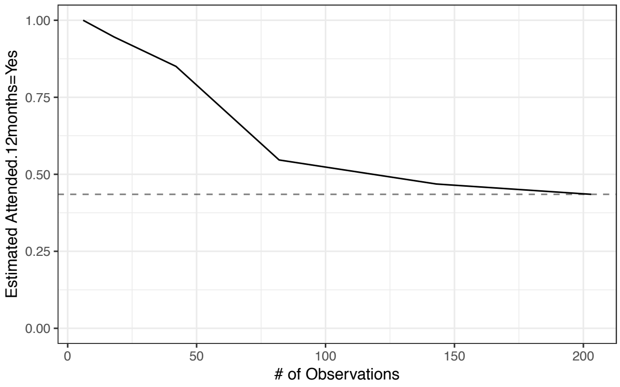

UOMM (Received Wristband)

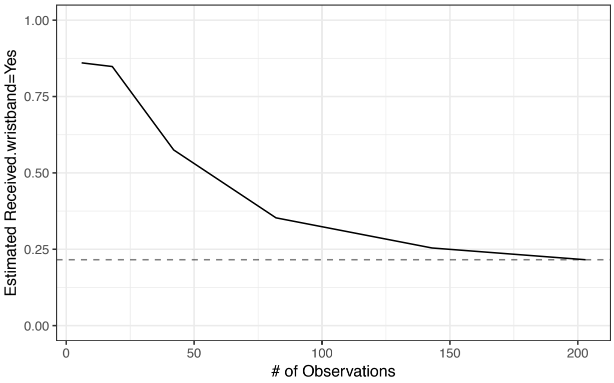

Site 8

SMM (Attended Sisters)

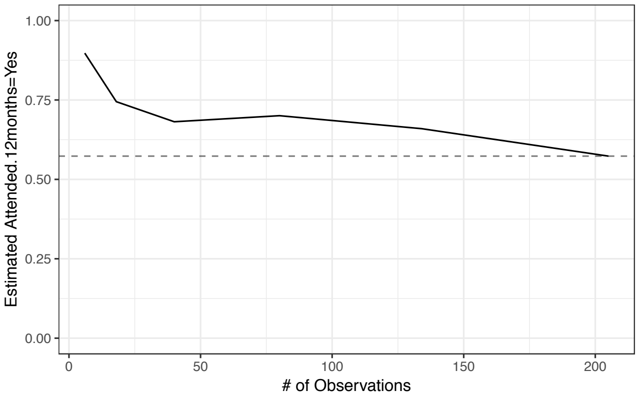

UOMM (Received Wristband)

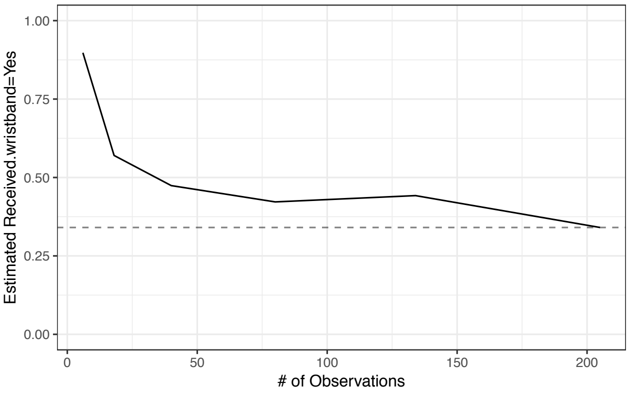

Site 9

SMM (Attended Sisters)

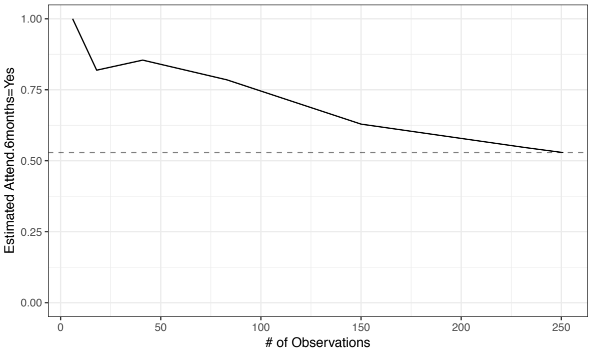

Site 10

SMM (Attended Sisters)

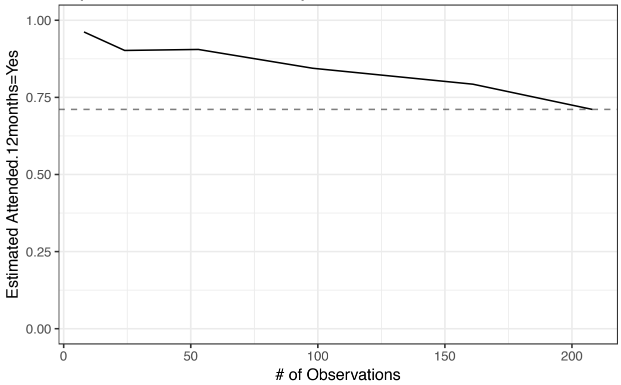

UOMM (Received Wristband)

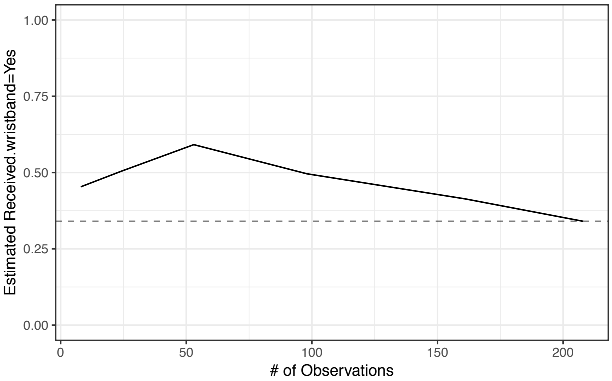

Site 11

UOMM (Received Wristband)

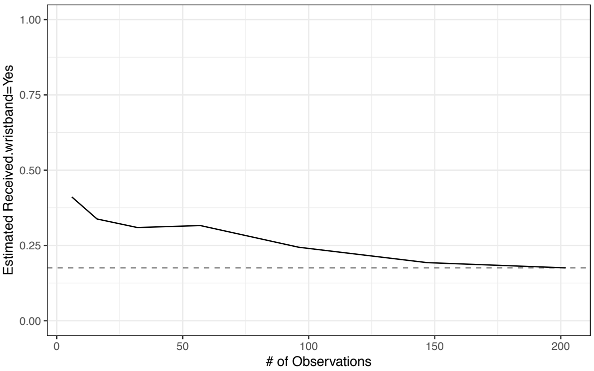

Site 12

SMM (Attended Sisters)

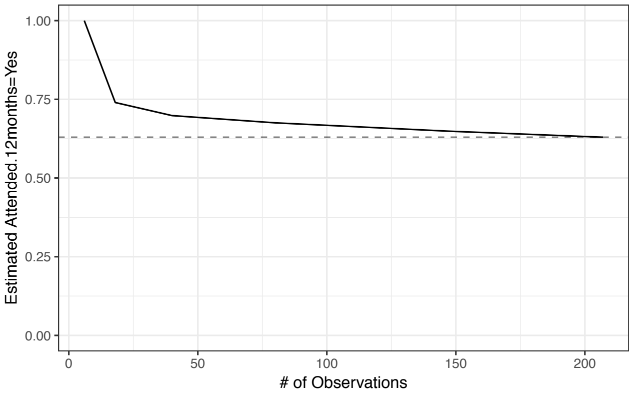

UOMM (Received Wristband)

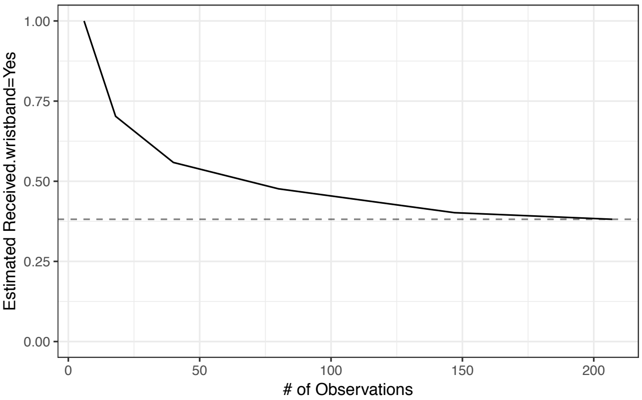

Site 13

SMM (Attended Sisters)

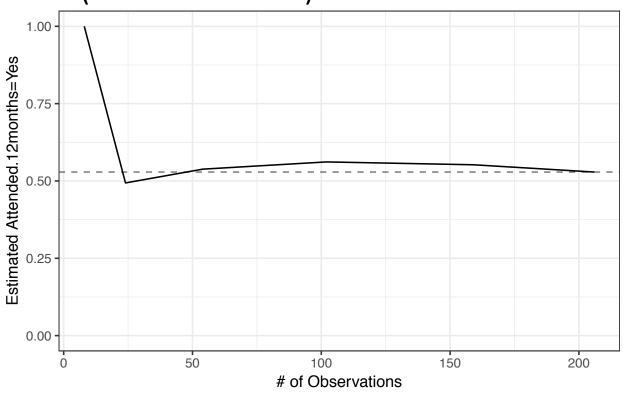

UOMM (Received Wristband)

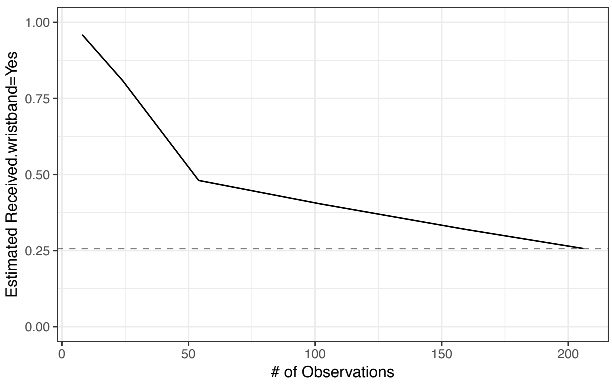

Site 14

SMM (Attended Sisters)

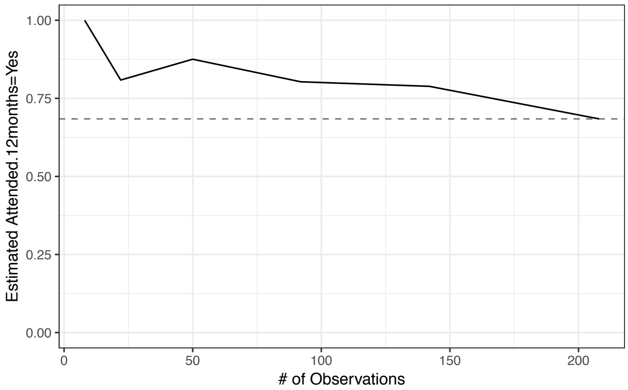

UOMM (Received Wristband)

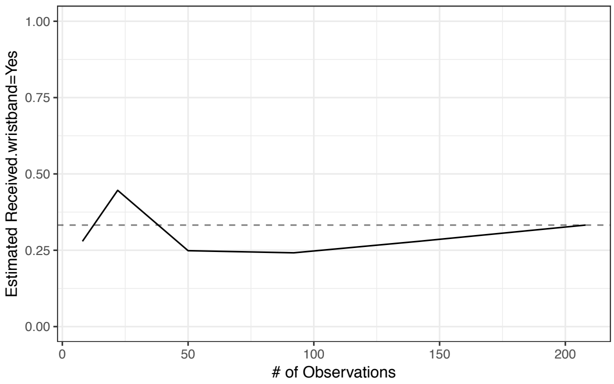

Site 15

SMM (Attended Sisters)

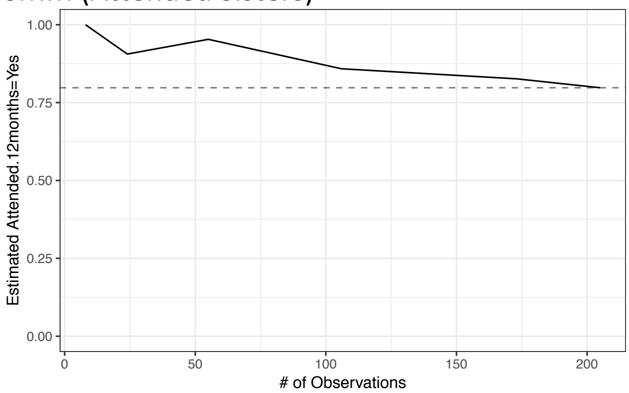

UOMM (Received Wristband)

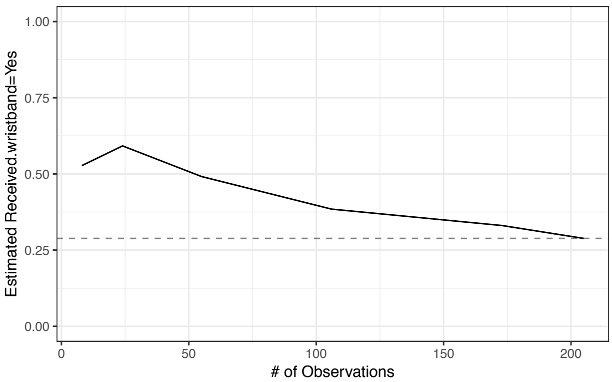

Site 16

SMM (Attended Sisters)

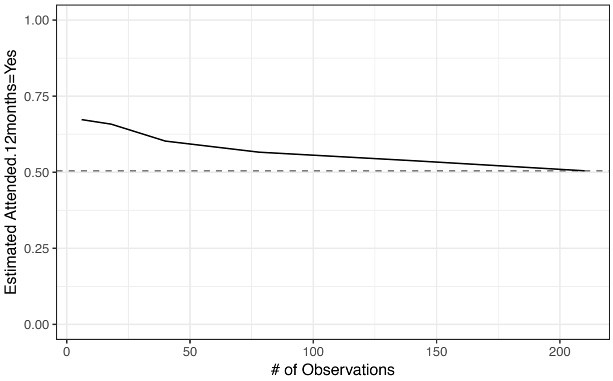

UOMM (Received Wristband)

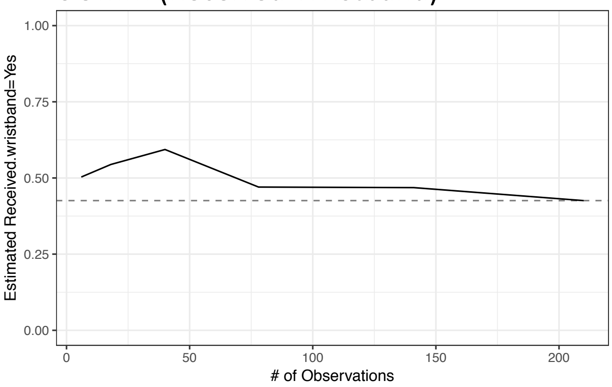

Site 17

SMM (Attended Sisters)

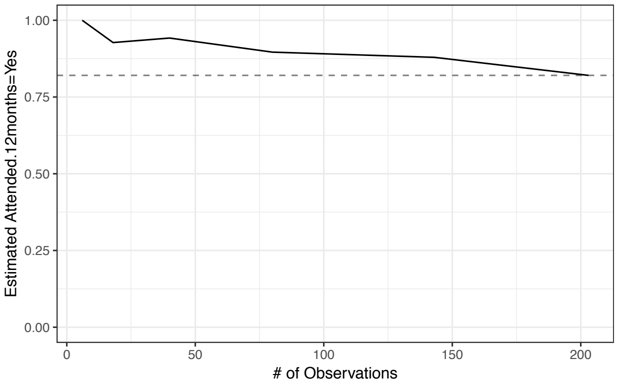

UOMM (Received Wristband)

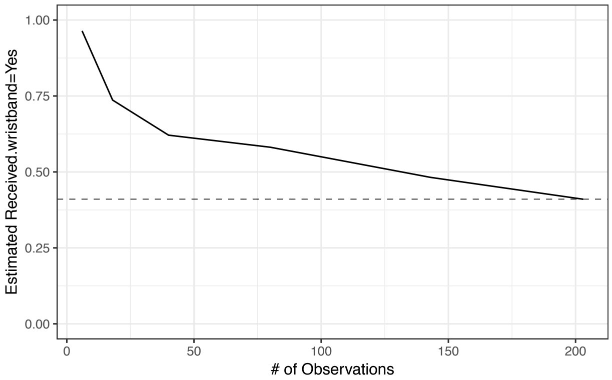

Site 18

SMM (Attended Sisters)

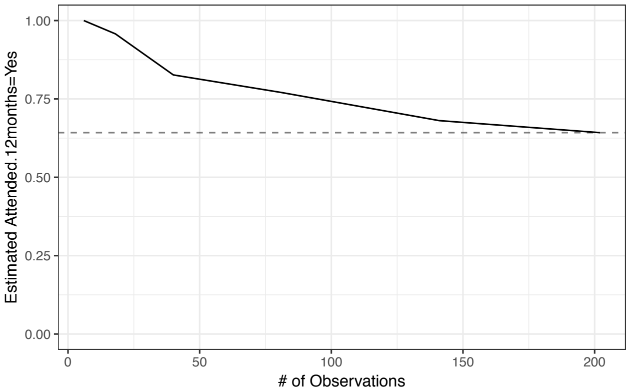

UOMM (Received Wristband)

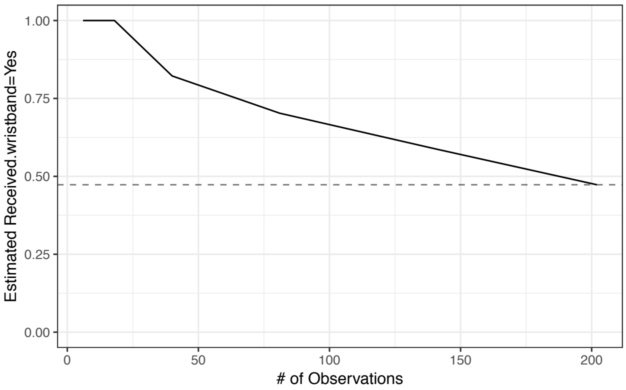

Site 19

SMM (Attended Sisters)

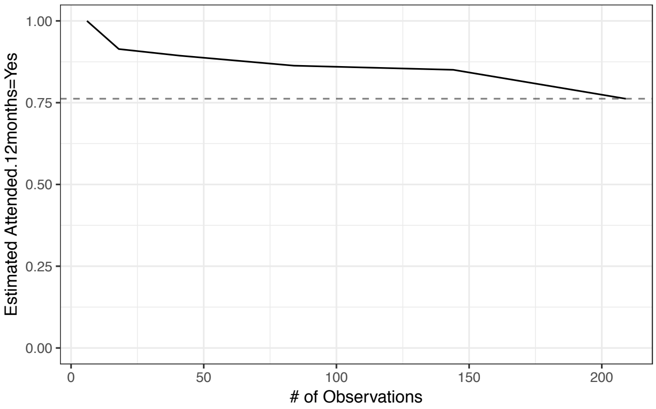

UOMM (Received Wristband)

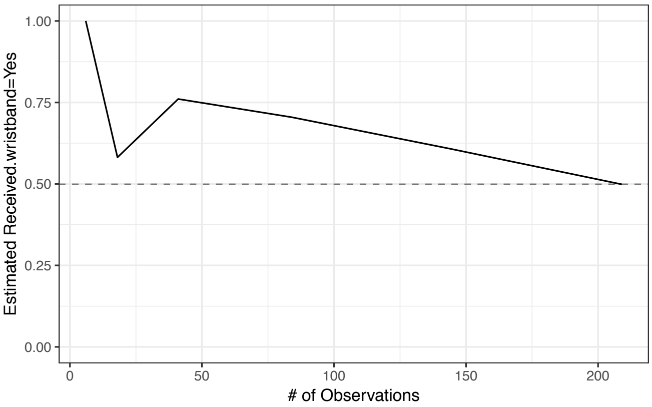

Table 1: Assessment of Estimators from RDS Surveys used in the Service Multiplier and Unique Object Multiplier Site PSE Methods

	SMM			UOMM		
Site	Convergence of Attended Clinic Estimator	Recruitment homophily by attendance at clinic	Differential activity: ratio of mean degree of those attending to not attending	Convergence of Received Wristband estimator	Recruitment homophily by whether received a wristband	Differential activity: ratio of mean degree of those receiving to not receiving a wristband
1	Good convergence	1.10	1.35	n/a		
2	Good convergence	1.16	0.98	Good convergence	1.10	1.93
3	Estimate potentially high	1.14	1.22	n/a		
4	Good convergence	1.09	1.31	Satisfactory convergence	1.21	0.99
5	Satisfactory convergence	1.02	1.46	Good convergence	1.06	1.36
6	Good convergence	1.04	1.10	Estimate potentially high	1.30	1.08
8	Estimate potentially high	1.05	1.29	Estimate potentially high	1.27	1.00
9	Estimate potentially high	1.01	1.50	Estimate potentially high	1.13	0.97
9	Estimate potentially high	1.08	1.16	n/a		
10	Estimate potentially high	1.07	1.02	Estimate potentially high	1.21	1.09
11	n/a			Good convergence	1.01	1.39
12	Good convergence	1.09	1.20	Good convergence	1.28	1.19
13	Good convergence	1.00	1.02	Satisfactory convergence	1.25	0.91
14	Estimate potentially high	0.98	1.31	Good convergence	1.12	0.97
14	Satisfactory convergence	1.17	1.50	Satisfactory convergence	1.21	1.67
15	Satisfactory convergence	1.03	1.28	Estimate potentially high	1.20	1.43
16	Satisfactory convergence	1.13	1.21	Satisfactory convergence	1.40	0.91
17	Estimate potentially high	1.02	1.01	Estimate potentially high	1.40	1.16
18	Satisfactory convergence	1.17	1.22	Estimate potentially high	1.15	1.18
20	n/a			n/a		
	**Mean:**	1.07	1.23	**Mean:**	1.21	1.20

*Recruitment homophily refers to the tendency for participants in the survey to recruit others who are like themselves on the characteristics of interest. A measure of 1 refers to similarity as would be expected by chance, negative numbers to dissimilarity and measures > 1 to similarity.

1. Guidelines on Estimating the Size of Populations Most at Risk to HIV. Geneva: World Health Organisation and UNAIDS; 2010.
2. Gile KJ, Handcock MS. Respondent-Driven Sampling: An Assessment of Current Methodology. Sociol Methodol. 2010;40(1):285-327.
3. Gile KJ, Johnson LG, Salganik MJ. Diagnostics for respondent driven sampling. Journal of the Royal Statistical Society: Series A. 2014.
4. MS. H, IE. F, KJ. G. RDS: respondent-driven sampling, Version 0.7-8. Project home page at http://hpmrg.org/, http://cran.r-project.org/package=RDS.2016 [
5. R Core team. R: A language and environment for statistical computing. R Foundation for Statistical Computing, Vienna, Austria. URL http://www.r-project.org/. 2015.

Appendix 2: RDS Survey questions used for individual site unique object and service multiplier method estimates

Service Multiplier Method: Sisters with a Voice attendance

2016 Surveys

Have you ever heard of the Sisters with a Voice programme?

0 No

1 Yes

999 I don't wish to answer

In the past 12 months have you attended the Sisters with a Voice clinic?

0 No

1 Yes

999 I don't wish to answer

2015 and 2017 surveys

Have you ever heard of the Sisters with a Voice programme?

0 No

1 Yes

999 I don't wish to answer

In the past 6 months have you attended the Sisters with a Voice clinic?

0 No

1 Yes

999 I don't wish to answer

Unique Object Multiplier Methods Wristband receipt questions

Q No	Question	Responses	Instructions
A8a	Have you received a wrist band with a Sisters with a Voice logo since January 2017?	Yes (1)<br>No (0)<br>No but I have seen it with others (2)	If 1 go to A8b<br>SKIP: 0 or 2 go to A9
A8b	How many wrist bands have you received?	Number pad	If 1 go to A8c<br>If >2 go to A8f
A8c	Where did you receive the wrist band?	Site 1 [NAME REDACTED] (1)<br>Site 2 [NAME REDACTED] (2)<br>Site 3 [NAME REDACTED] (3)<br>Site 4 [NAME REDACTED] (4)	go to A8d
A8d	What month did you receive the wrist band?	January (1)<br>February (2)<br>March (3)<br>April (4)	go to A8e
A8e	What was the colour of the wrist band?	light blue (1)<br>navy blue (2)<br>pink (3)<br>orange (4)<br>green (5)<br>yellow (6)<br>red (7)<br>black (8)<br>other (9)<br>peach (10)<br>I don't know (88)<br>I don't wish to answer (999)	go to A9
A8f	Where did you receive the first wrist band?	Site 1 [NAME REDACTED] (1)<br>Site 2 [NAME REDACTED] (2)<br>Site 3 [NAME REDACTED] (3)<br>Site 4 [NAME REDACTED] (4)	go to A8g

A8g	What month did you receive the first wrist band?	January (1)<br>February (2)<br>March (3)<br>April (4)	go to A8h
A8h	What was the colour of the first wrist band?	light blue (1)<br>navy blue (2)<br>pink (3)<br>orange (4)<br>green (5)<br>yellow (6)<br>red (7)<br>black (8)<br>other (9)<br>peach (10)<br>I don't know (88)<br>I don't wish to answer (999)	go to A8i
A8i	Where did you receive the second wrist band?	Site 1 [NAME REDACTED] (1)<br>Site 2 [NAME REDACTED] (2)<br>Site 3 [NAME REDACTED] (3)<br>Site 4 [NAME REDACTED] (4)	go to A8j
A8j	What month did you receive the second wrist band?	January (1)<br>February (2)<br>March (3)<br>April (4)	go to A8k
A8k	What was the colour of the second wrist band?	light blue (1)<br>navy blue (2)<br>pink (3)<br>orange (4)<br>green (5)<br>yellow (6)<br>red (7)<br>black (8)<br>other (9)<br>peach (10)<br>I don't know (88)<br>I don't wish to answer (999)	go to A9

Appendix 3: Workshop Outline

Population size estimation: overview and approaches to developing an estimate of the female sex worker population in Zimbabwe
June 6th and 7th, 2017
Centre for Sexual Health and HIV/AIDS Research (CeSHHAR), Harare, Zimbabwe

Objectives

1. To review methods to estimate population sizes of marginalised populations such as female sex workers
2. To review female sex worker population size estimates (PSEs) for 20 sites across Zimbabwe
3. To use a facilitated process to discuss and agree methods for developing a national estimate of the number of female sex workers in Zimbabwe

Participants

Participants included programmers working with the Sisters with a Voice female sex worker programme run by CeSHHAR Zimbabwe, which provides peer education, outreach, community mobilisation and clinical services to sex workers at 36 sites around Zimbabwe; experts in Zimbabwe HIV epidemiology from across government agencies, international agencies, programmes and funders; and researchers (epidemiologists, statisticians) from CeSHHAR Zimbabwe and the Measurement and Surveillance of HIV Epidemics (MeSH) Consortium Key Populations Working Group.

Facilitators:
Frances M Cowan – Director, CeSHHAR Zimbabwe, Liverpool School of Tropical Medicine (LSTM) UK
Elizabeth Fearon- Epidemiologist, MeSH Consortium Key Populations Working Group, London School of Hygiene and Tropical Medicine (LSHTM) UK
Sungai T Chabata – Statistician, CeSHHAR Zimbabwe
Attendees:
Samson Chidiya – HIV Prevention Specialist, USAID Zimbabwe
Trust Chiguvare – Public Health Specialist Monitoring and Evaluation, CDC Zimbabwe
Elizabeth Gonese – Public Health Specialist HIV Surveillance, CDC Zimbabwe
Sitholubuhle Magutshwa – Social Scientist with Sisters with a Voice programme, CeSHHAR Zimbabwe
Victor Makaza – Monitoring and Evaluation Officer, National AIDS Council Zimbabwe
Matthews Maruva – Senior M&E Specialist, USAID Zimbabwe
Absolom Masendeke – RTI International, Zimbabwe
Tendai Mhaka – Key Populations Coordinator, National AIDS Council Zimbabwe
Mutsa Mhangara – USAID Zimbabwe
Tendayi Ndori-Mharadze – Programme Director: Key Populations, Manager of Sisters with a Voice Programme, CeSHHAR Zimbabwe
Brilliant Nkomo – Strategic Information Coordinator, Ministry of Health and Child Care Zimbabwe
Isaac Taramusi – Monitoring and Evaluation Coordinator, National AIDS Council Zimbabwe

Agenda

Day 1: Overview of Population Size Estimation
Morning
- Introductions and objectives
- Overview of Population Size Estimation: rationale, approaches, key issues

Afternoon
- PSE's of female sex workers at sites across Zimbabwe

Day 2: PSE for female sex workers in Zimbabwe and extrapolation to the national level
Morning
- Planning a Population Size Estimation Study
- Overview of extrapolation methodologies
- Developing a national PSE of female sex workers in Zimbabwe:
    - Workshop

Afternoon
- Developing a national PSE of female sex workers in Zimbabwe:
    - Workshop
- Next steps and ways forward

Key decisions made:
- How to summarise the size estimate for the twenty sites with direct PSEs where multiple methods were used
- Approach to extrapolating from these 20 sites to obtain a national population size estimate
- Develop a list of likely hotspot sites for female sex work around Zimbabwe. These were decided to be all those 36 sites with Sisters clinics, plus any additional sites estimated to have concentrations of female sex workers by the workshop attendees.
- Matching hotspots into strata of likely similarity of SW prevalence
- Estimate of the proportion of all female sex workers in Zimbabwe who would be found in one of the hotspot sites.

Methods

The process for reaching decisions during the workshop was as follows. First, workshop attendees attended training on methods used to estimate population sizes, their strengths, weaknesses and uncertainties, estimates obtained from 20 sites in Zimbabwe, and methods used to extrapolate site estimates to the national level. Background and contextual information behind each decision was given, followed by an open discussion amongst all workshop attendees. The facilitators then asked participants to put forward suggestions- these were each discussed and debated, a consensus amongst workshop attendees was reached and participants were reminded of the decisions reached at regular intervals and at the end of the workshop. Particular attention was given to each participant's particular background and expertise.

When considering additional sites that could be hotspots for FSW around Zimbabwe, participants considered each province in turn and reviewed a map. Not all suggestions put forward were necessarily accepted by the group. When creating strata of hotspots considered to be similar to each other with respect to the likely prevalence of sex work among adult women (unknown for those sites without direct PSEs), a table of site classifications was projected so that participants could consult it. This included site names, provinces, primary and secondary classifications (often related to main economic industry: mining, tourism, border site, farming, fishing, army base, mining, rural growth-point) and male and female population from Census 2012. After a first list of groupings was reached, participants were asked to discuss and agree the groupings again before the groupings were finalised.

Attachments:
Slides used during the workshop (excluding those with names sites other than Harare and Bulawayo, decided to be too sensitive to report without anonymising sites) are included in Appendix 4.

MeSH Consortium
Measurement & Surveillance of HIV Epidemics

CeSHHAR Zimbabwe

Population Size Estimation

June 6th and 7th, 2017
Harare

Elizabeth Fearon, LSHTM
Sungai Chabata, CeSHHAR Zimbabwe
Frances Cowan, CeSHHAR Zimbabwe

www.mesh-consortium.org.uk

Agenda

Day 1: Overview of Population Size Estimation

Morning

- Introductions and objectives
- Overview of Population Size Estimation: rationale, approaches, key issues

Afternoon

- PSE’s of female sex workers in Zimbabwe
- Planning a Population Size Estimation Study exercise

Agenda

Day 2: PSE for female sex workers in Zimbabwe and extrapolation to the national level

- Overview of extrapolation methodologies

- Developing a national PSE of female sex workers in Zimbabwe:
  - Introduction
  - Workshop
- Next steps and ways forward

MeSH Consortium
Measurement & Surveillance of HIV Epidemics

Introductions

- Name, organisation, key responsibilities
- Why interested in population size estimation- how does it relate to your work?
- Review Agenda- anything to add?

MeSH Consortium
Measurement & Surveillance of HIV Epidemics

MeSH Consortium
Measurement & Surveillance of HIV Epidemics

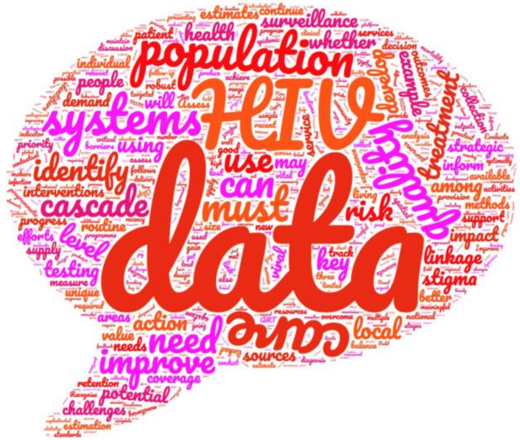

The ***Measurement and Surveillance of HIV Epidemics (MeSH) Consortium*** strengthens data systems, data analysis, and data use by HIV programmes to drive programme improvement and enhanced strategic information to increase prevention and treatment coverage among target populations and accelerate the decline of HIV incidence in sub-Saharan Africa

www.mesh-consortium.org.uk

BILL & MELINDA GATES foundation

MeSH Consortium
Measurement & Surveillance of HIV Epidemics

Partners

α – network
Analysing Longitudinal Population-based HIV/AIDS data on Africa

HIV Modelling Consortium

CDC
Centers for Disease Control and Prevention

UCSF
University of California San Francisco
advancing health worldwide

LINKAGES
Across the Continuum of HIV Services for Key Populations

The University of North Carolina at Chapel Hill

University of Cape Town · Universiteit van Kaapstad · iYunivesithi yaseKapa

SPES BONA

UHURU NA UMOJA

!KE E: /XARRA //KE

CeSHHAR Zimbabwe

SEARCH
Sustainable Evaluation through Analysis of Routinely Collected HIV data

Investing in our future
The Global Fund
To Fight AIDS, Tuberculosis and Malaria

Primary Health Care
A Healthy Nation
Ministry of Health and Child Welfare
Zimbabwe

United States Agency International Development
USAID

IUPUI
Richard M. Fairbanks School of Public Health
Indiana University
Indianapolis

HARAMBEE

Johns Hopkins
Bloomberg School of Public Health

Bill & Melinda Gates foundation

u^b
Universität Bern

Joint United Nations Programme on HIV/AIDS
UNAIDS
UNHCR·UNICEF·WFP·UNDP·UNFPA
UNODC·ILO·UNESCO·WHO·WORLD BANK

London School of Hygiene & Tropical Medicine

World Health Organization

NASTAD
National Alliance of State & Territorial AIDS Directors

Unity · Freedom · Work

IeDEA

rbc
Rwanda Biomedical Center
A Healthy People. A Wealthy Nation

MeSH Cor
Measurement & Surveillance of HIV Epidemics

Partners

UCSF
University of California
San Francisco
advancing health worldwide

THE UNIVERSITY
of NORTH CAROLINA
at CHAPEL HILL

University of Cape Town · iYunivesithi yaseKapa · Universiteit van Kaapstad

JOHNS HOPKINS
BLOOMBERG SCHOOL
of PUBLIC HEALTH

u[b]
UNIVERSITÄT
BERN

LONDON
SCHOOL *of*
HYGIENE
&TROPICAL
MEDICINE

MeSH Consortium
Measurement & Surveillance
of HIV Epidemics

MeSH structure

Mortality

- How to estimate mortality among people commencing HIV antiretroviral therapy?
- How to estimate HIV attributable mortality in the general population?

Case-based surveillance

- Are systems in place that are context appropriate, feasible, scalable, and sustainable for CBS in SSA?
- How to establish and conduct HIV CBS in SSA?

Key Populations

- How to optimise extrapolation and triangulation for population size, prevalence, and incidence estimation?
- How to measure HIV prevention cascades?
- How to characterise metrics of stigma to track stigma reduction?

Guidelines & dissemination

- Care & prevention cascade training
- Guideline development
- Agenda setting and dissemination

MeSH Consortium
Measurement & Surveillance of HIV Epidemics

Working Group 3: among key populations estimate size and location, prevention and treatment coverage, and prevalence and incidence

- Extrapolation and Triangulation

  Use observed direct estimates plus contextual data (available from all areas) to learn about areas without direct estimates

- Prevention cascades
- Metrics to track progress on stigma

MeSH Consortium
Measurement & Surveillance of HIV Epidemics

Introductions

Name, organisation, role

Why interested in population size estimation- how does it relate to your work?

Review Agenda- anything to add?

- Do you currently use estimates of the size of key populations in your work?
- Where do these estimates come from?
- How much do you trust these estimates? How important is it that the estimates are very accurate?

Population Size Estimation: Overview

MeSH Consortium

Measurement & Surveillance of HIV Epidemics

What populations are we talking about?

HIV risk context

Do not appear in a census or difficult to sample

- Problems with self-reporting -> social stigma, marginalised populations, criminalised populations
- Mobile populations, across geographies
- Mobile definition, across time (eg moving in and out of periods of risk)

MeSH Consortium
Measurement & Surveillance of HIV Epidemics

Why do we want to estimate the number of sex workers at sites, regions and countries?

- Service delivery targeting and planning
  - What populations are at high risk of HIV?
  - Where do services need to be located?
- Programme monitoring (‘denominator’)
  - Provide a ‘denominator’ for services uptake
  - Use routinely collected data to estimate programme coverage and evaluate reach
- Epidemic prediction
  - Input estimates into models such as SPECTRUM
- Allocation of funding
- Advocacy

MeSH Consortium
Measurement & Surveillance of HIV Epidemics

Methods for Population Size Estimation

- Literature review
- Mapping methods: census and enumeration
- Multiplier methods
- Population survey methods
- New approaches

MeSH Consortium
Measurement & Surveillance of HIV Epidemics

Methods for Population Size Estimation

- Literature review
- Mapping methods: census and enumeration
- Multiplier methods
- Population survey methods
- New approaches

No 'gold standard'

MeSH Consortium
Measurement & Surveillance of HIV Epidemics

Methods: Literature Search

Review estimates from other sites, likely range

Convert to prevalence

Multiply by population size

Estimates of the number of female sex workers in different regions of the world

J Vandepitte, R Lyerla, G Dallabetta, F Crabbé, M Alary, A Buvé

Sex Transm Infect 2006;**82**(Suppl III):iii18–iii25. doi: 10.1136/sti.2006.020081

See end of article for authors' affiliations

Correspondence to: Dr J Vandepitte, STD/HIV Research and Intervention Unit, Institute of Tropical Medicine, Nationalestraat 155, 2000 Antwerp, Belgium; jvdpitte@itg.be

Accepted for publication 18 April 2006

Objectives: To collect estimated numbers of female sex workers (FSW) and present proportions of FSW in the female population (FSW prevalence) in different regions of the world.
Methods: Subnational and national estimated numbers of FSW reported in published and unpublished literature, as well as from field investigators involved in research or interventions targeted at FSW, were collected. The proportion of FSW in the adult female population was calculated. Subnational estimates were extrapolated to national estimates if appropriate. Population surveys were scanned for proportions of adult women having sex in exchange for money or goods.
Results: In sub-Saharan Africa, the FSW prevalence in the capitals ranged between 0.7% and 4.3% and in other urban areas between 0.4% and 4.3%. Population surveys from this same region yielded even higher proportions of women involved in transactional sex. The national FSW prevalence in Asia ranged between 0.2% and 2.6%; in the ex-Russian Federation between 0.1% and 1.5%; in East Europe between 0.4% and 1.4%; in West Europe between 0.1% and 1.4%; and in Latin America between 0.2% and 7.4%. Estimates from rural areas were only available from one country.
Conclusions: Although it is well known and accepted that FSW are a highly vulnerable group in the scope of the HIV epidemic, most countries in the world do not know the size of this population group. The estimates of the prevalence of FSW presented in this paper show how important this hard-to-reach population group is in all parts of the world.

In many countries, the HIV epidemic is concentrated in subgroups of the population whose behaviour exposes them to a high risk of acquiring HIV infection. These subgroups include injecting drug users, men who have sex with men, and commercial sex workers—female and male. Numerous studies have documented significantly higher [illegible]

outside of known venues for sex work. Therefore they are even more difficult to reach than women known as direct sex workers.[2–10] As a consequence, the absolute size of the FSW population remains largely unknown.

In many countries data are available on HIV prevalence among sex workers. However in estimating national HIV [illegible]

MeSH Consortium
Measurement & Surveillance of HIV Epidemics

Methods: Wisdom of the crowd

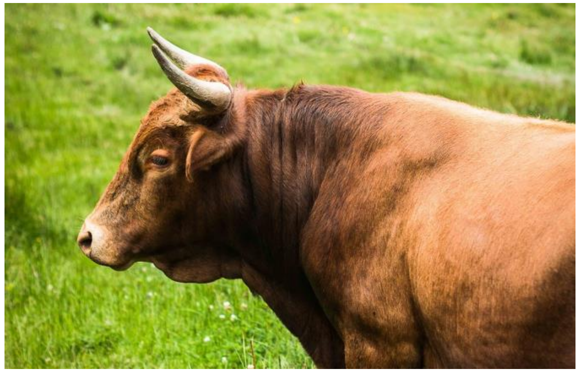

How many female sex workers do you think there are in this town?

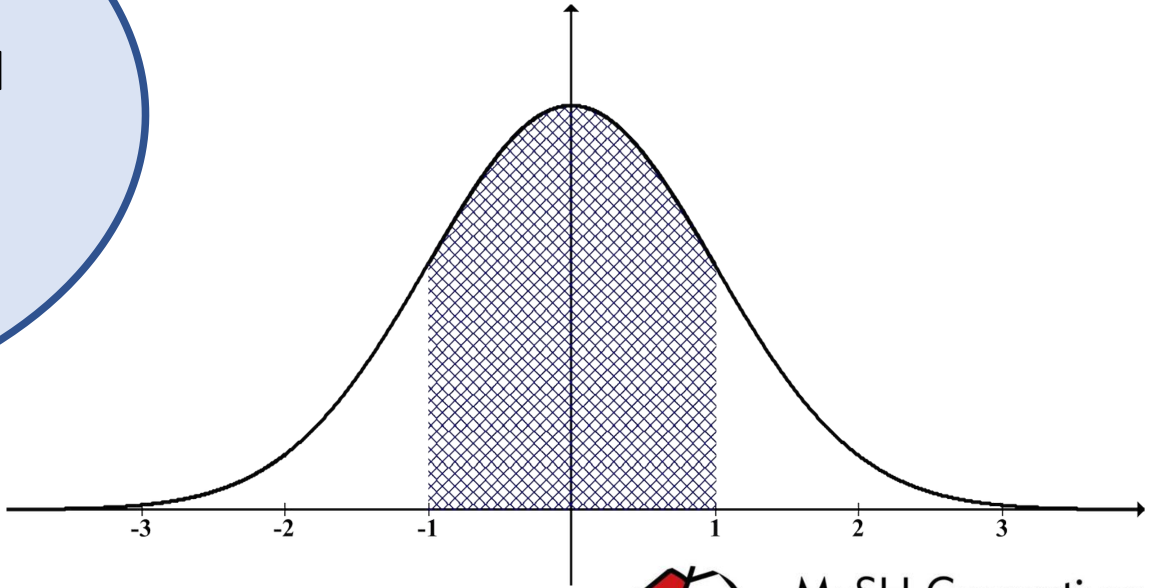

MeSH Consortium
Measurement & Surveillance of HIV Epidemics

Methods: Wisdom of the crowd

Strengths

- Easily added to a survey
- Special knowledge
- If ask a large group, less likely to be affected by outliers

Weaknesses

- Everyone might have the same tendency to be wrong (systematic bias)
- Think about size of site
- Segregated population

MeSH Consortium
Measurement & Surveillance of HIV Epidemics

Mapping Methods: Census and Enumeration

Construct map of where the population congregates

- Key informants, peer educators, focus groups

Census: use map as a tool to count ALL members of the population

Enumeration: use map as a sampling frame to select venues; count all of the population in the sampled venues and extrapolate to all venues.

- Stratify venues, apply averages to strata
- Time/place

MeSH Consortium
Measurement & Surveillance of HIV Epidemics

Mapping Methods: Census and Enumeration

Strengths

- Provides a minimum number
- Easy to understand and communicate
- Good check alongside other methods

Weaknesses

- Miss members of population not at these venues
- Need to consider time/space
- Very time intensive for large sites

MeSH Consortium
Measurement & Surveillance of HIV Epidemics

Methods: Multiplier Methods

Uses two sources of data

1. Register or count of the target population (received service, visited programme, received token) -> **M**
2. Representative survey of the target population -> **P**

$$N=M/P$$

MeSH Consortium
Measurement & Surveillance of HIV Epidemics

Service Multiplier Method

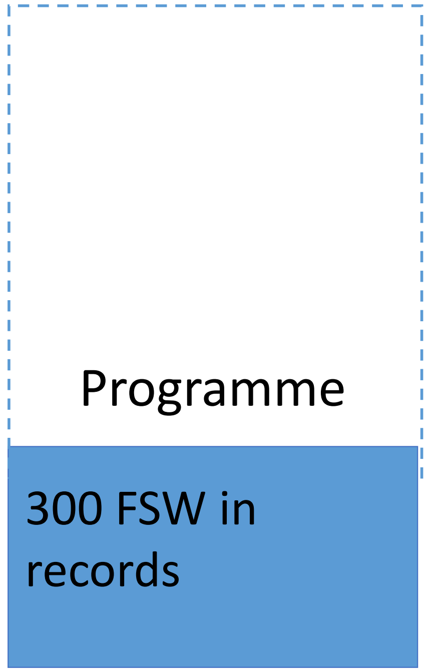

MeSH Consortium
Measurement & Surveillance of HIV Epidemics

Service Multiplier Method

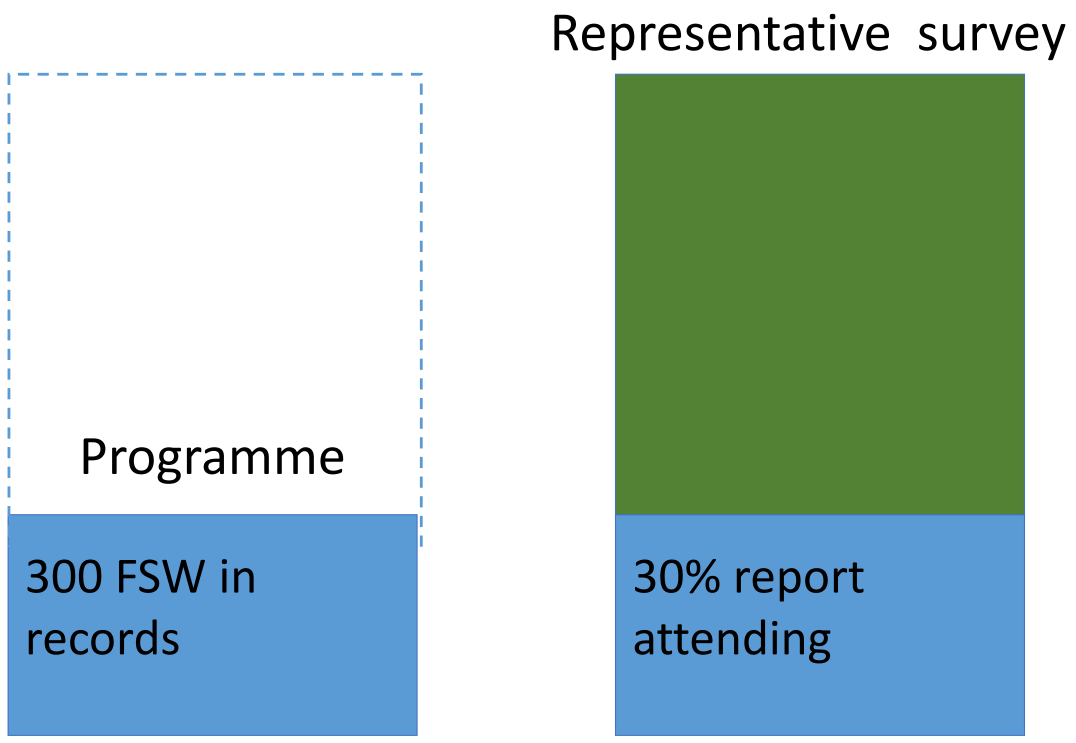

MeSH Consortium
Measurement & Surveillance of HIV Epidemics

Service Multiplier Method

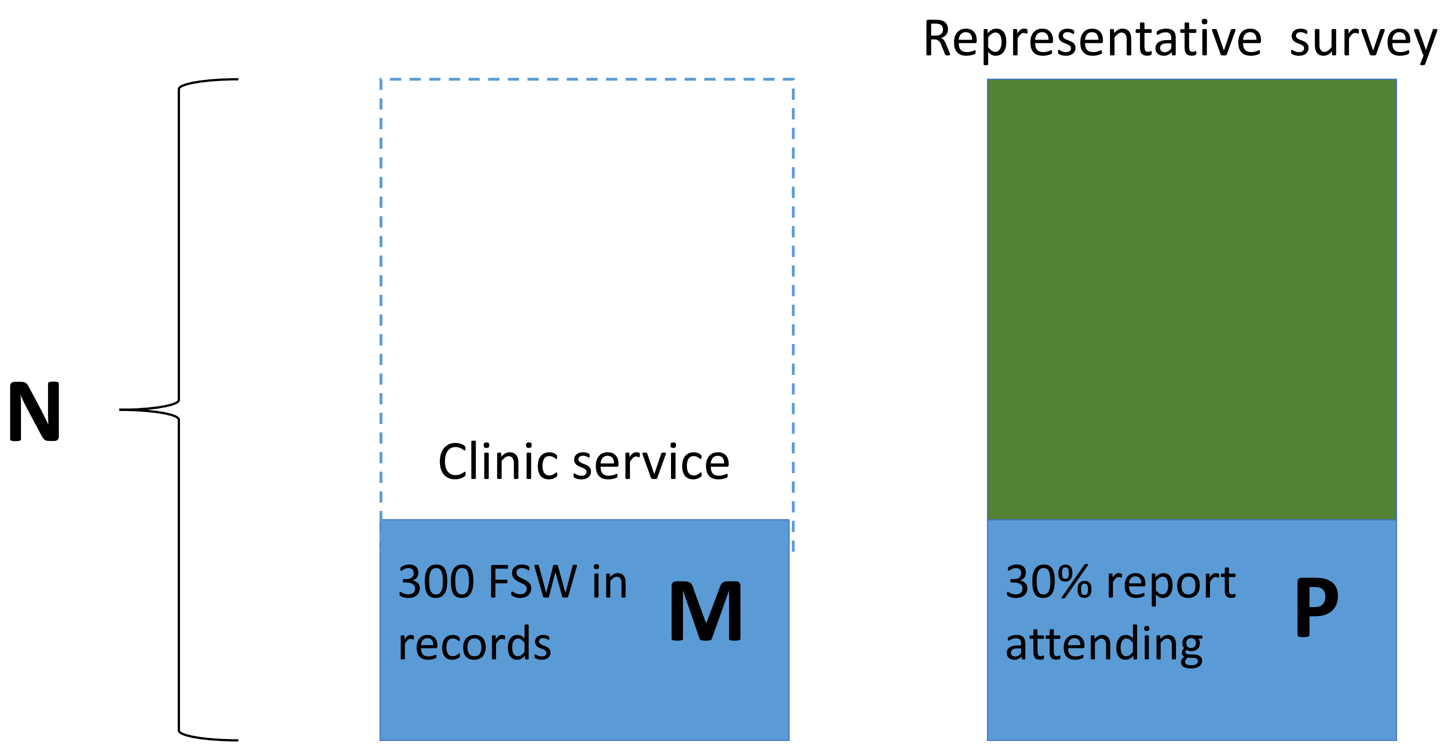

N=M/P 1000 = 300* 1/0.3

MeSH Consortium
Measurement & Surveillance of HIV Epidemics

‘Unique Object’ Multiplier Method

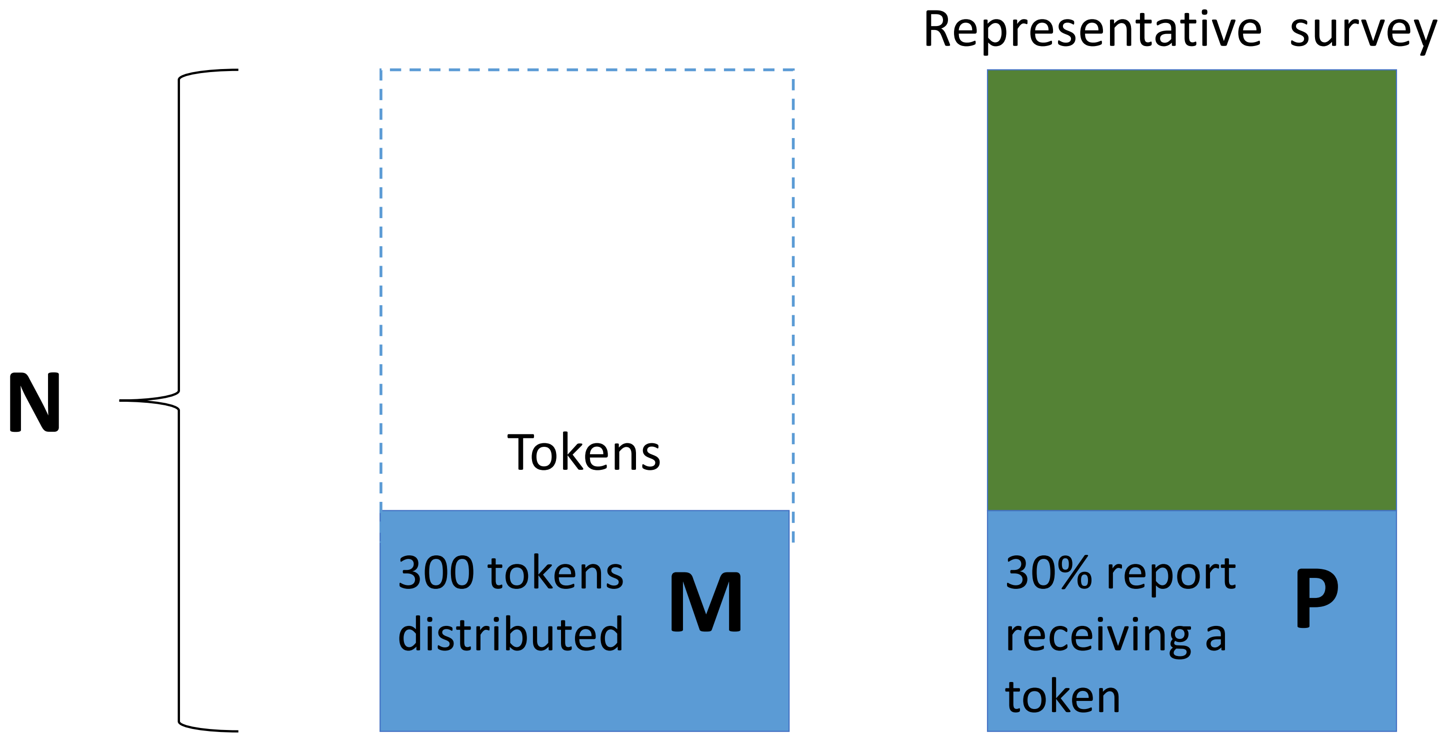

MeSH Consortium
Measurement & Surveillance of HIV Epidemics

Multiplier Method: Other multipliers

- Membership of community groups, social clubs
- Membership of social networking sites (if can get list)
  - Online dating profiles
  - Facebook groups
  - What's App groups
- Attendance at population-specific events
- Arrest data (where applicable)

MeSH Consortium
Measurement & Surveillance of HIV Epidemics

Multiplier Method: Assumptions

1. All members of the population being counted should have a **non-zero probability of being included** in both data sources.
2. Individuals should **not be counted more than once** in each data source.
3. The two data sources should be **independent** of each other.
4. The representative data source should be a **random sample** of the target population.

MeSH Consortium
Measurement & Surveillance of HIV Epidemics

Multiplier Method

Strengths

- Simple calculation
- Use existing service data
- Survey questions relatively easy/low burden

Weaknesses

- Can give very variable results
- High uncertainty
- Independence of data sources challenging in practice
- Inconsistencies in population definition

MeSH Consortium
Measurement & Surveillance of HIV Epidemics

Methods: Capture Re-Capture

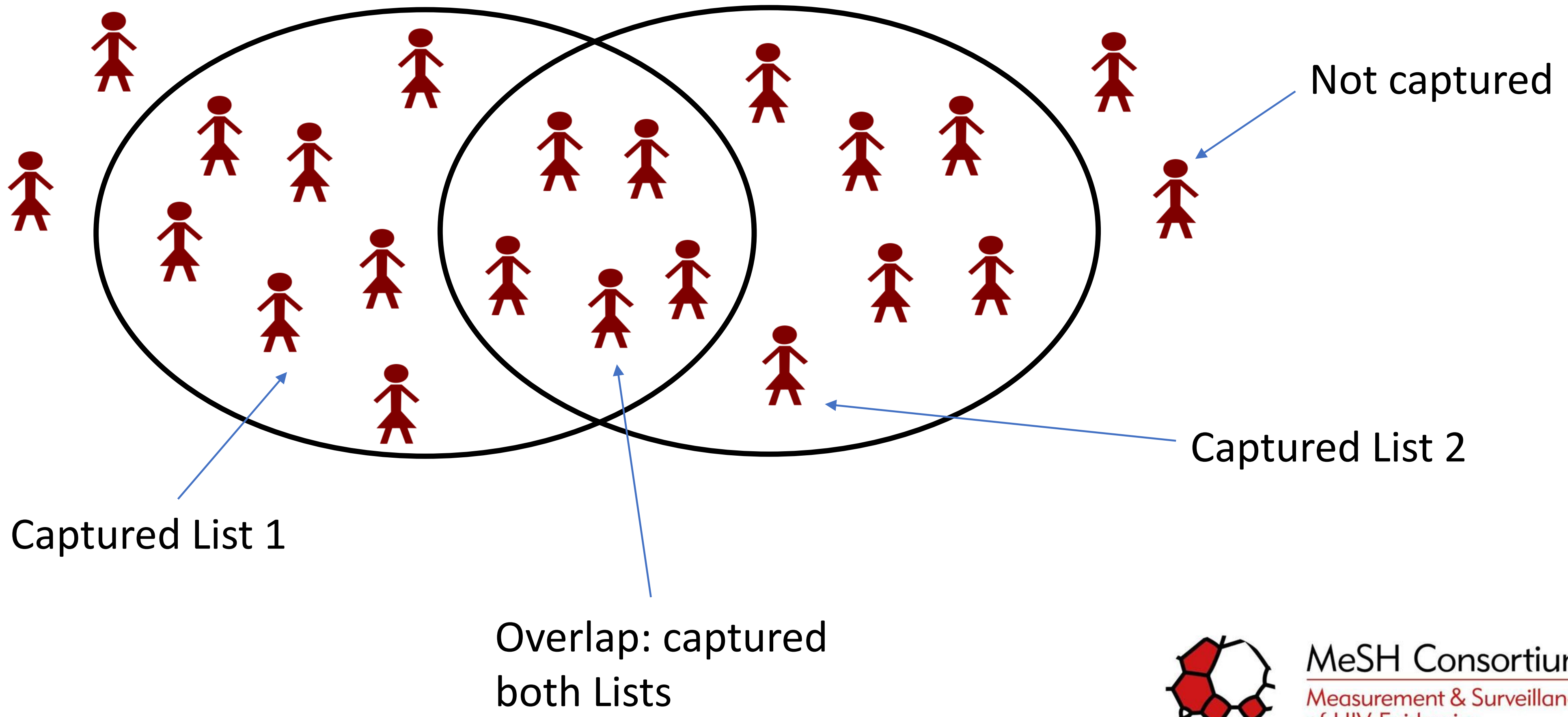

MeSH Consortium
Measurement & Surveillance of HIV Epidemics

Methods: Capture Re-Capture

$$N = \frac{\text{Captured List 1 x Captured List 2}}{\text{Overlap Captured both lists}}$$

MeSH Consortium
Measurement & Surveillance of HIV Epidemics

Methods: Capture Re-Capture

Strengths

- Simple calculation
- Less time-intensive than census and enumeration
- No representative survey required
- Recommend multiple captures

Weaknesses

- Misses those not present at venues
- Difficult to meet assumptions in practice:
  - Two ‘captures’ must be independent
  - Identify individuals accurately

MeSH Consortium
Measurement & Surveillance of HIV Epidemics

Population Surveys: asking about behaviours

- Question to define population in representative population survey
  - Lifetime behaviour
  - Recent behaviour

MeSH Consortium
Measurement & Surveillance of HIV Epidemics

Population Surveys: asking about behaviours

Strengths

- Provides estimates at different levels (given power) or at least a framework for extrapolation
- Provides other information about the population

Weaknesses

- Statistical power
- Social desirability bias
- Question to define population
- Likelihood of inclusion in survey
- Time (ever/now)

MeSH Consortium
Measurement & Surveillance of HIV Epidemics

Population Surveys: Network Scale-Up

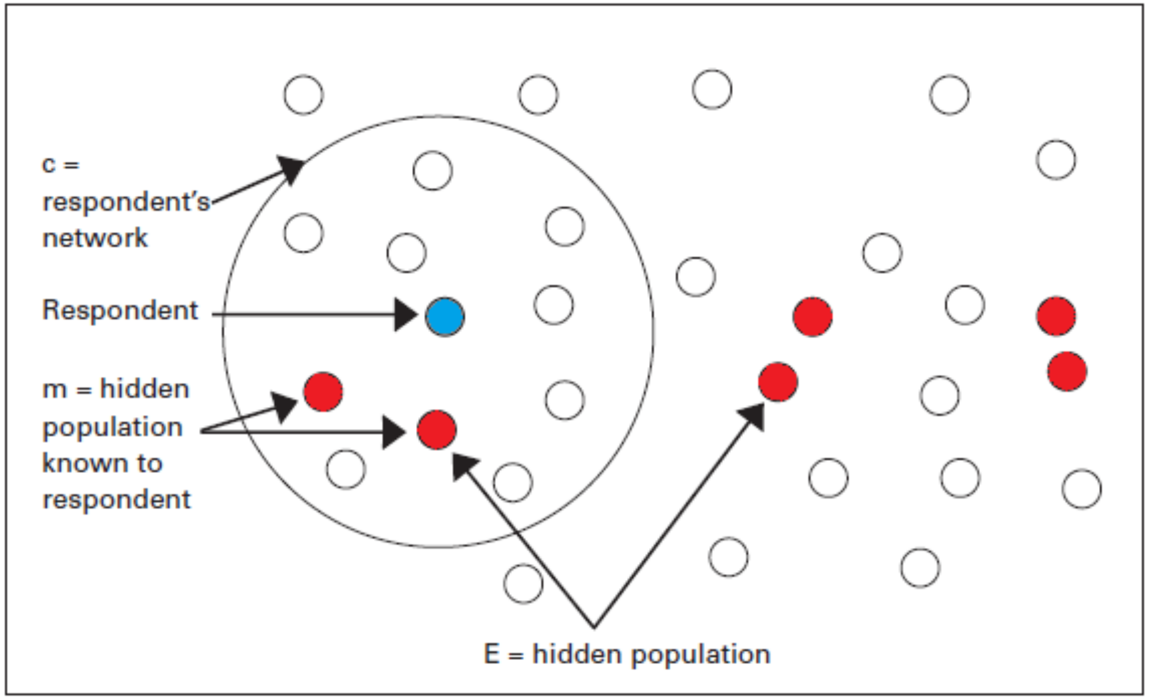

Shokoohi M, Baneshi MR, Haghdoost AA. Size Estimation of Groups at High Risk of HIV/AIDS using Network Scale Up in Kerman, Iran. Int J Prev Med. 2012;3(7):471-6.

Proportion of hidden population members in the total population can be estimated from the proportion within the personal networks of a random sample of the population.

$$m/c = e/t$$

Obtainable from a population survey.

MeSH Consortium
Measurement & Surveillance of HIV Epidemics

Network Scale-Up Assumptions

- Members of population are known to be members
  No transmission bias

- Equal network size between members and non-members
  No **popularity bias**

- Random mixing or even distribution of populations
  No **barrier effect**

- Reporting is accurate
  No **reporting bias**

MeSH Consortium
Measurement & Surveillance of HIV Epidemics

Network Scale-Up

Strengths

- Improved ‘sample size’
- No self-report
- Methods in development, lots of interest

Weaknesses

- Assumptions difficult to meet
- Personal network size difficult to measure
- Transmission bias likely-> ‘Game of Contacts’

MeSH Consortium
Measurement & Surveillance of HIV Epidemics

Methods: RDS-Successive Sampling

- Based on theoretical decline in network size over sampling wave
- Imputed 'network visibility'
- Bayesian framework

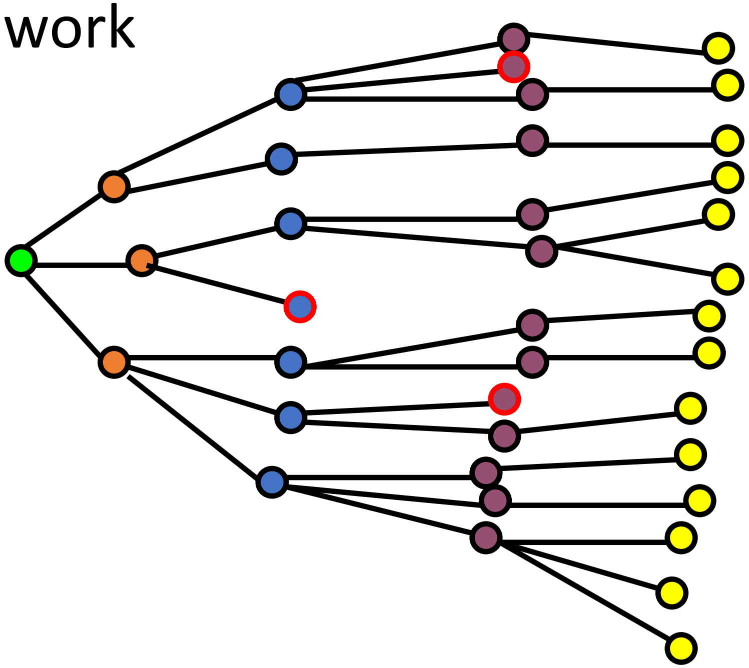

MeSH Consortium
Measurement & Surveillance of HIV Epidemics

RDS-Successive Sampling

Strengths

- Requires only an RDS survey (though better with additional estimates to compare, inform prior)
- Framework for synthesising estimates?

Weaknesses

- Assumptions about degree decay over sample waves
- New method: little tested in practice so far

Issues with population size estimation

- No gold standard method
- All methods prone to bias
- Widely variable depending on method
- Definition of the population
- Fluctuations over time: migrations, moving in and out of risk
- Extrapolation to different level (usually site to national level)
- Difficulty assessing time trends

MeSH Consortium
Measurement & Surveillance of HIV Epidemics

High variability

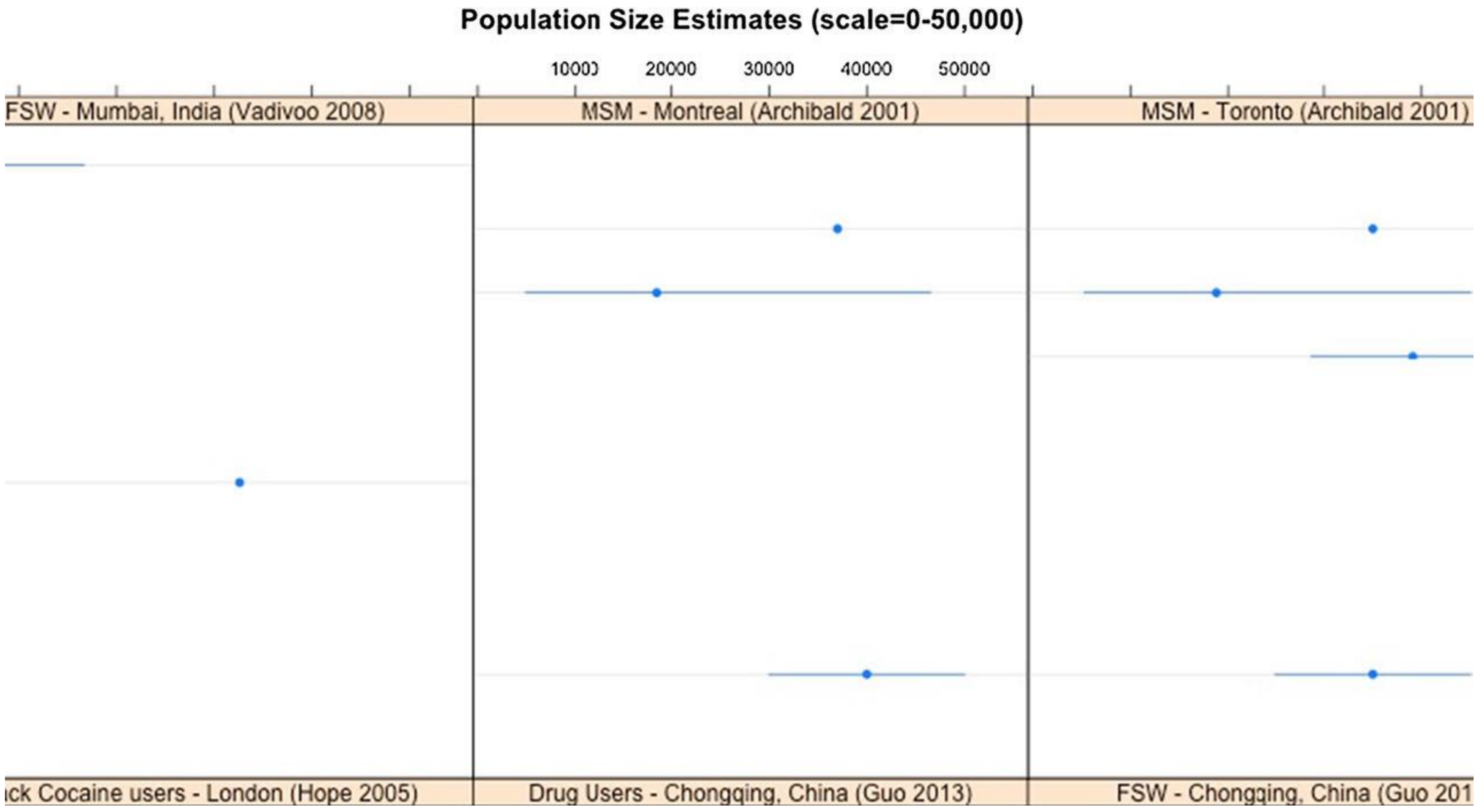

Wesson P, Reingold A, McFarland W. Theoretical and Empirical Comparisons of Methods to Estimate the Size of Hard-to-Reach Populations: A Systematic Review. AIDS Behav. 2017.

MeSH Consortium
Measurement & Surveillance of HIV Epidemics

Sources of uncertainty

- Random error: wide confidence intervals
  - Sample size calculations
- Assumptions of methods difficult to meet in practice -> biases
  - Some assumptions can be investigated (Chabata et al)
- Combining estimates from different methods
- Extrapolation method or lack thereof

→ lack of certainty should be reflected in how PSE's are used

MeSH Consortium
Measurement & Surveillance of HIV Epidemics

Systematic biases by method

- Systematic review found 341 published KP size estimates, 25 had multiple methods for side by side comparison
- No evidence of “best” method, all could be biased up or down by theory or observation
  - Literature tends to the middle
  - Wisdom of the crowd tends to be low
  - Delphi tends to the middle
  - Mapping estimates tend to be low
  - Multiplier methods and capture-recapture estimates can be wildly high or low

Wesson P, Reingold A, McFarland W. Theoretical and Empirical Comparisons of Methods to Estimate the Size of Hard-to-Reach Populations: A Systematic Review. AIDS Behav. 2017.

MeSH Consortium
Measurement & Surveillance of HIV Epidemics

Triangulation and Synthesis

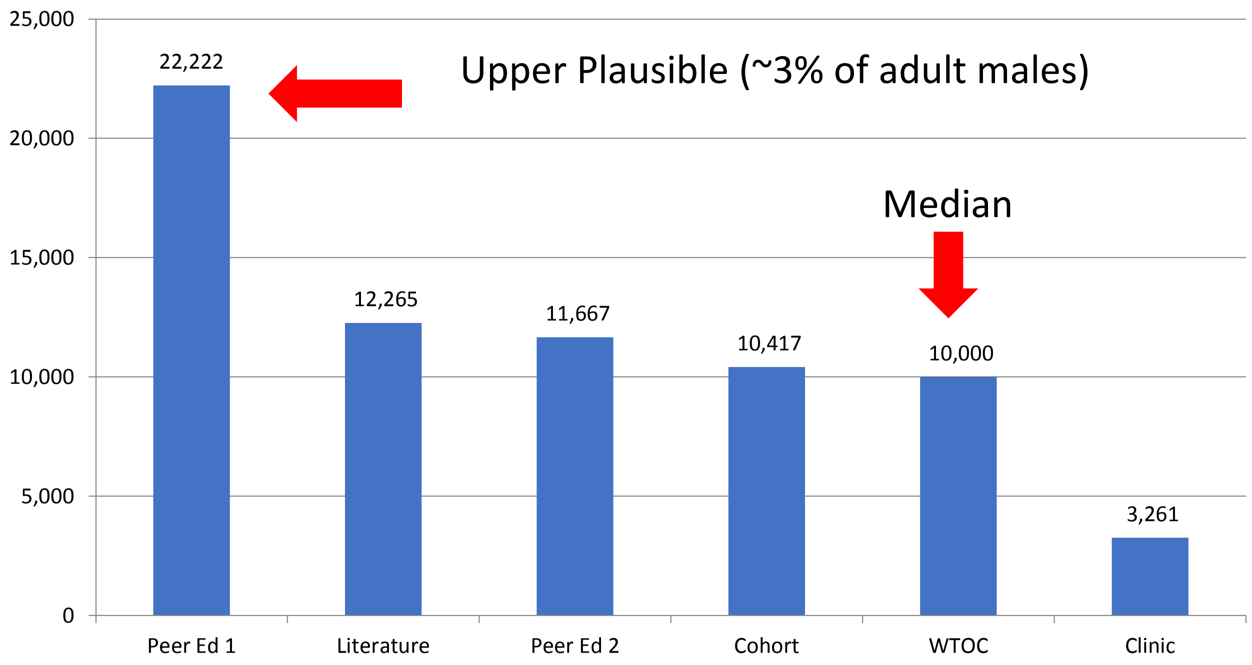

MSM population size estimates, median and plausibility bounds, Nairobi, 2010.

Okal J, Geibel S, Muraguri N, Musyoki H, Tun W, Broz D, et al. Estimates of the size of key populations at risk for HIV infection: men who have sex with men, female sex workers and injecting drug users in Nairobi, Kenya. Sex Transm Infect. 2013;89(5):366-71

MeSH Consortium
Measurement & Surveillance of HIV Epidemics

Plausibility

- Minimum estimates
  - Census
  - Population Service data (eg attendance at sex worker's clinic)
- Maximum estimates

- Comparison to other settings

- Relative plausibility: Site A versus Site B

MeSH Consortium
Measurement & Surveillance of HIV Epidemics

Recommendations

For those conducting size estimations:

- Use multiple methods, triangulation
- Sample size calculations for survey-based methods (this afternoon)
- Use and explain a principled method for extrapolation (tomorrow)

For those using population size estimates:

Use upper and lower plausibility bounds- do not rely on point estimate

MeSH Consortium
Measurement & Surveillance of HIV Epidemics

Discussion

Do you think the way in which population size estimates are used currently reflects the uncertainty around them?

MeSH Consortium
Measurement & Surveillance of HIV Epidemics

MeSH Consortium

Measurement & Surveillance of HIV Epidemics

MeSH Consortium

Measurement & Surveillance of HIV Epidemics

Planning a Population Size Estimation Study

www.mesh-consortium.org.uk

Who is the population?

- Behaviours versus people
- Periods of risk
- Place/mobility questions
- Seasonality

Defining your site

- What are the boundaries?
- Does your population reside there permanently or temporarily?
- Do you intend to update findings again in the future?
- Do you intend to use your findings to extrapolate to another site?

MeSH Consortium
Measurement & Surveillance of HIV Epidemics

Choosing appropriate methods

- What are the estimates for?
- Characteristics of the population- venued based? Well ‘networked’?
- Existing data, resources for new data collection
- Size of site(s)
- Levels of representativeness (small truck stop, town, city, country)

MeSH Consortium
Measurement & Surveillance of HIV Epidemics

Stakeholder involvement

- Who are the estimates of benefit to?

- Who can help you understand your population?

  - Representatives from your population of interest
  - National/regional government
  - Programme planners, service providers, NGO’s
  - Others with data

MeSH Consortium
Measurement & Surveillance of HIV Epidemics

Ethics

- Vulnerable, often criminalised populations
- Security around mapping
- Identification via unique objects, RDS coupons
- Publication of site names and numbers? (Feedback)
- Benefit to whom?

Communication of Findings

- Incentives to choose higher or lower figures
- Ethics and communication of findings
- How to communicate uncertainty in estimates

MeSH Consortium
Measurement & Surveillance of HIV Epidemics

Sample Size Calculations

- Decisions about surveys
  - Population-based surveys
  - Target population surveys: Respondent Driven Sampling, Time/Location
- Decisions about multipliers
  - (eg no. unique objects distributed)
- Decisions about captures
- Mapping decisions, sites within strata
- Extrapolate to regional/national level estimate?

MeSH Consortium
Measurement & Surveillance of HIV Epidemics

Sample Size for Multiplier Method Studies with RDS

- Important to obtain estimates with reasonable precision for study aims (random variation), lack of current guidance
- Estimate P (proportion using service) using RDS survey, (N=M/P)
- Higher variance in RDS surveys than in a simple random sample survey, Design Effects (DEFF's) of 2-4 or higher
- Using Delta method combining variance in M and P, estimate effect of sample size on the width of 95% Confidence interval for different assumed values of M and P

Fearon E, Chabata S, Thompson J, Cowan FM, Hargreaves JR. Sample size calculations for population size estimation studies using multiplier methods with respondent driven sampling surveys. Submitted 2017.

MeSH Consortium
Measurement & Surveillance of HIV Epidemics

Example: Sample Size for PSE of female sex workers in Harare

Table 1: Number of FSW attending the Sisters programme and effect on P, given the total population of FSW 15,000 in Harare (mid-point estimate based on literature, Vandepitte 2006)

Reference Period, to April 23, 2015	Number of Unique FSW Visiting, M	Estimated P, assuming population = 15,000
**1 month**	85	0.006
**3 months**	560	0.037
**6 months**	952	0.063
**12 months**	1542	0.103
**24 months**	2227	0.148

Sisters programme clinic visit data up to April 2015

MeSH Consortium
Measurement & Surveillance of HIV Epidemics

Figure 1: Effect of reference period on P, width of the 95% CI around the PSE and sample size required for estimating the number of FSW in Harare

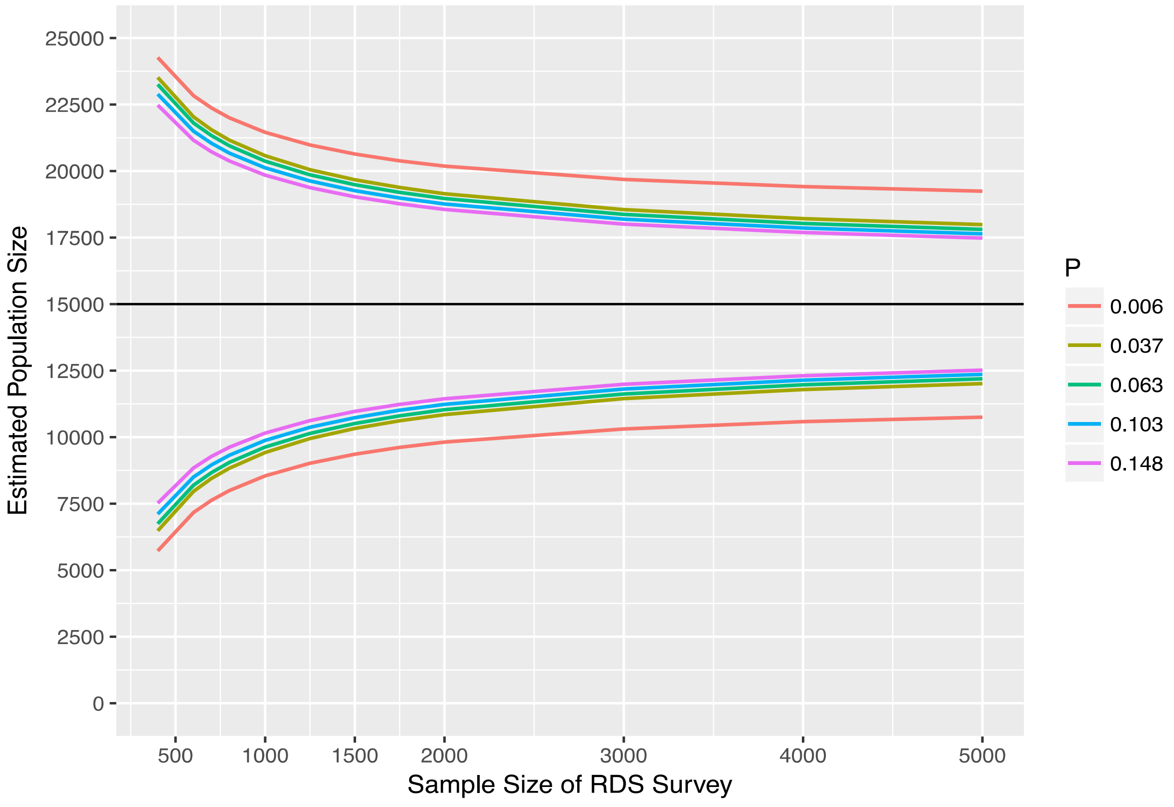

Fearon E, Chabata S, Thompson J, Cowan FM, Hargreaves JR. Sample size calculations for population size estimation studies using multiplier methods with respondent driven sampling surveys. Submitted 2017.

MeSH Consortium
Measurement & Surveillance of HIV Epidemics

MeSH Consortium

Measurement & Surveillance of HIV Epidemics

MeSH Consortium
Measurement & Surveillance of HIV Epidemics

CeSHHAR Zimbabwe

Developing a National Estimate of the Number of Female Sex Workers in Zimbabwe

www.mesh-consortium.org.uk

Approaches to Extrapolation in other settings

- Assume exchangeability: prevalence from sampled sites = prevalence non-sampled sites
- Stratification: urban/rural, regional, type of sites (tourist, mining, truck-stop, etc)
- Matching
- Regression-based approaches
  - Model to correlate FSW prevalence with other district co-variates
  - Model for district sampling probabilities
  - Bayesian models

MeSH Consortium
Measurement & Surveillance of HIV Epidemics

Review of PSE's for Key Populations in LMIC's 2010-2016

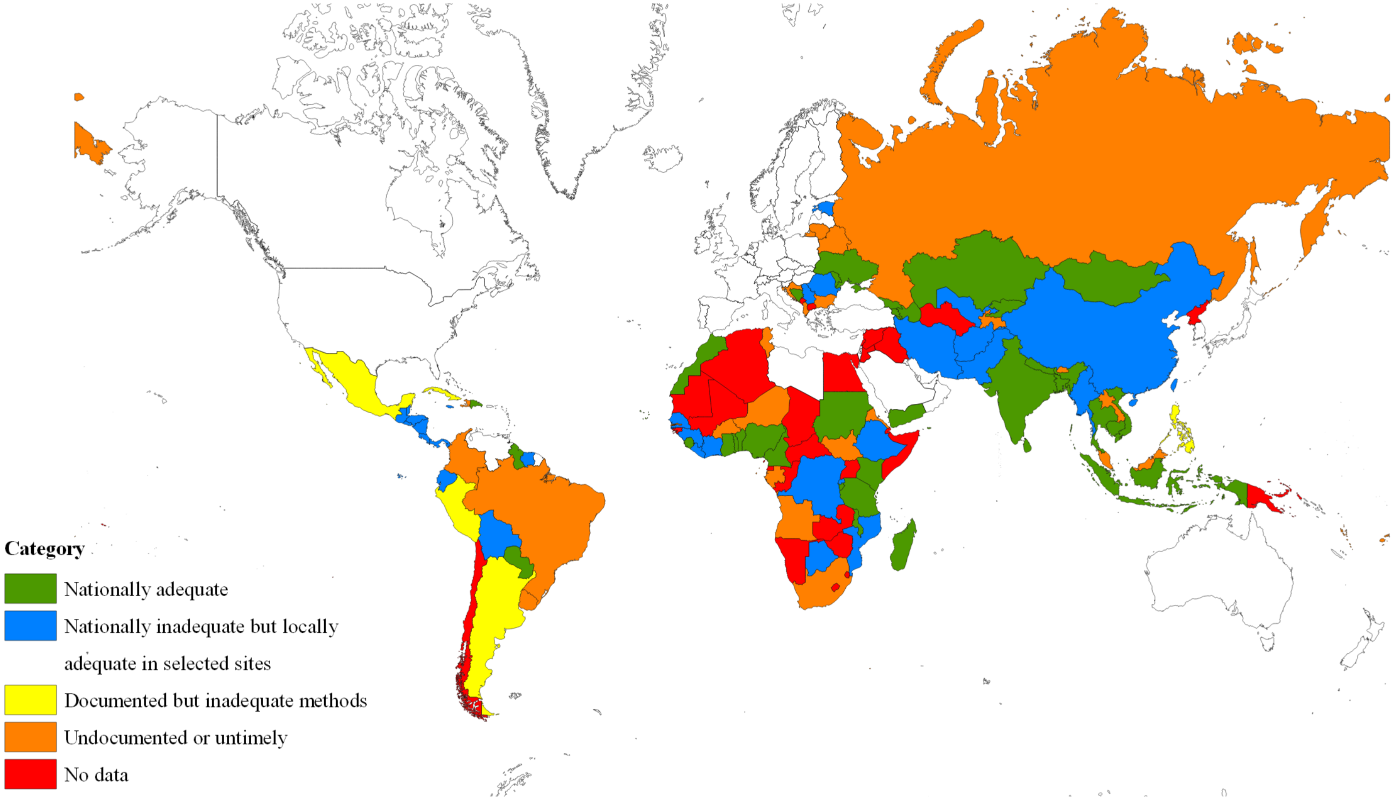

Fig 1. Categorization of population size estimates of female sex workers, men who have sex with men, people who inject drugs, and transgender women in low- and middle-income countries, 2010–2014.

Sabin K, Zhao J, Garcia Calleja JM, Sheng Y, Arias Garcia S, Reinisch A, et al. Availability and Quality of Size Estimations of Female Sex Workers, Men Who Have Sex with Men, People Who Inject Drugs and Transgender Women in Low- and Middle-Income Countries. PLoS One. 2016;11(5):e0155150.

MeSH Consortium
Measurement & Surveillance of HIV Epidemics

1. **Nationally adequate:**
   - PSE’s derived using 1) multiplier; 2) capture –recapture; 3)mapping/enumeration; 4)Network scale-up or population survey; 5)RDS-SS
   - National-level estimates or extrapolated from multiple sites with a “clear approach” to extrapolation
   - Two key population groups
2. **Nationally inadequate but locally adequate in selected cities**
   - PSE’s derived using 1) multiplier; 2) capture –recapture; 3)mapping/enumeration; 4)Network scale-up or population survey; 5)RDS-SS
   - Estimates are only from sites where targeted programs are avail- able but are insufficient for national program use.
   - Two key population groups
3. **Documented estimates but inadequate methods**
   - estimatesarederivedfrom1)expert opinions; 2) Delphi; 3) wisdom of crowds; 4) programmatic results or registry or 5) regional benchmarks.
   - Estimates may or may not be national
4. **Undocumented or untimely:** estimates are reported but not documented or were derived prior to 2010.
5. **No data:** no size estimates are reported

MeSH Consortium
Measurement & Surveillance of HIV Epidemics

Table 2. Application of methods in estimating population size estimates for female sex workers (FSW), men who have sex with men (MSM), people who inject drug (PWID) and transgender women in low- and middle-income countries, 2010–2014.

Methods	FSW	MSM	PWID	Transgender women
Multiplier	29	31	23	2
Capture re-capture	19	17	11	2
Census & enumeration	19	10	3	1
Programmatic mapping	27	23	12	9
Network scale up method or population-based survey	4	11	9	
RDS-SS successive sampling	2	3	2	
Administrative registry/programmatic results	2	1	1	1
Regional benchmark	3	7	2	1
Population-based survey	1	5	4	
Expert opinion (wisdom of crowds/literature/ Delphi/key informants)	13	10	7	2
Wisdom of crowds	6	10	3	
Not Reported	9	6	6	2
Total number of countries	87	88	53	17

Sabin K, Zhao J, Garcia Calleja JM, Sheng Y, Arias Garcia S, Reinisch A, et al. Availability and Quality of Size Estimations of Female Sex Workers, Men Who Have Sex with Men, People Who Inject Drugs and Transgender Women in Low- and Middle-Income Countries. PLoS One. 2016;11(5):e0155150.

MeSH Consortium
Measurement & Surveillance of HIV Epidemics

Classification of extrapolation approaches, Sabin et al 2016

1. Proportion of adult males or females: national estimates are calculated based on a proportion or a range of proportions of adult males or females, who are key population community members.
2. Summed up: national estimate is the sum of site-specific estimates with no adjustment.
3. Regression or probability formula: regression models were used to estimate populations in areas without an estimation exercise using information from those areas where estimations are available.
4. Based on one selected number: national estimate is extrapolated based on one estimate among a number of estimated numbers.
5. Delphi or consensus: a formal process considering different factors to arrive at an estimate.
6. No extrapolation.

MeSH Consortium
Measurement & Surveillance of HIV Epidemics

Table 5. Approaches used for extrapolations to national population size estimates in countries with known estimation methods for female sex workers (FSW), men who have sex with men (MSM), people who inject drug (PWID) and transgender women in low- and middle-income countries, 2010–2014.

Approaches for extrapolations	FSW	MSM	PWID	Transgender women
Proportion of adult population	21	30	10	4
Based-on one selected estimate	4	2	4	
Summed up from site-specific results	8	7	4	1
Regression or models	9	13	6	4
Delphi/consensus	6	5	5	1
Total number of countries with extrapolations	58	57	29	10

Sabin K, Zhao J, Garcia Calleja JM, Sheng Y, Arias Garcia S, Reinisch A, et al. Availability and Quality of Size Estimations of Female Sex Workers, Men Who Have Sex with Men, People Who Inject Drugs and Transgender Women in Low- and Middle-Income Countries. PLoS One. 2016;11(5):e0155150.

MeSH Consortium
Measurement & Surveillance of HIV Epidemics

Information in Zimbabwe to develop a National PSE for Female Sex Workers

We have a lot of high quality data:

- 20 sites with direct PSE’s, most with more than one method (+RDS surveys and programme data)
- 16 sites with in-depth programme data
- Coverage across the country
- 2012 Census
- National surveys, ZIMPHIA

How shall we use this to develop a national PSE?

MeSH Consortium
Measurement & Surveillance of HIV Epidemics
